# Progress, challenges, and lessons for climate resilient and low carbon health systemsin Latin America and the Caribbean: an exploratory mixed-methods study

**DOI:** 10.11606/s1518-8787.2026060007430

**Published:** 2026-07-06

**Authors:** Yasna Palmeiro-Silva, Camila Llerena-Cayo, Sebastian Bauhoff, Kristie L. Ebi, Catharina Giudice, Cristian A. Herrera, Jeremy J. Hess, Zuhelen Padilla, Karen Alicia Polson, Zoila Vela-Clavo, Daniel Forsin Buss, Stella M. Hartinger

**Affiliations:** 1 University of Washington. Center for Health and the Global Environment. Seattle, WA, United States; 2 University College London. Institute for Global Health. London, United Kingdom; 3 Universidad Peruana Cayetano Heredia. Centro Latino Americano de Excelencia en Cambio Climático y Salud. Lima, Peru; 4 Inter-American Development Bank. Washington, DC, United States; 5 Boston University School of Medicine. Department of Emergency Medicine. Boston, MA, United States; 6 World Bank, Health Nutrition and Population Global Practice. Santiago, Chile; 7 Pan American Health Organization. Washington, DC, United States

**Keywords:** Climate, Climate Change, Health, Health Systems, Environmental Health

## Abstract

**OBJECTIVE:**

To assess progress, challenges, and enabling factors for building climate-resilient and low-carbon health systems across Latin America and the Caribbean, a region facing accelerating climate-sensitive health burdens amidst persistent health system fragilities.

**METHODS:**

We conducted an explanatory, sequential, mixed-methods study integrating quantitative analysis of the Pan American Health Organisation Climate Change and Health surveys from 2021/2022 (n = 24 countries) and 2023/2024 (n = 27 countries) with semi-structured interviews involving four countries demonstrating progress (Argentina, Chile, Jamaica, Peru). Quantitative data were analysed descriptively across three sub-regions (Caribbean, Central America, South America). Qualitative data underwent two-stage coding (deductive and inductive) with three-researcher consensus to identify barriers, enablers, and lessons learned.

**RESULTS:**

By 2023/2024, 93% of countries had designated climate-health focal points (71% in 2021/2022). However, implementation gaps persist: less than 50% of countries had integrated climate change into national health reports; 22%–40% developed national climate-health strategies; and vulnerability assessments rarely informed policy. Access to international climate finance remained inequitable. Whilst 60%–74% developed disaster preparedness plans, only 30%–44% implemented public health communication campaigns. Training focused on environmental health personnel, with doctors, nurses, and planning staff minimally engaged. Qualitative analysis revealed interconnected barriers: climate change perceived as distant rather than urgent, competing priorities overwhelming decision-makers, institutional silos, and misalignment between available training and local needs. Key enablers included linking climate action to established health priorities, institutionalising responsibilities through formal mechanisms, multi-stakeholder engagement, and committed individuals with diplomatic skills navigating cross-sectoral dynamics.

**CONCLUSION:**

Latin America and the Caribbean countries are establishing foundations for climate-resilient and low-carbon health systems, but translating governance progress into sustained implementation requires addressing systemic barriers through institutionalisation beyond political cycles, tailored capacity building, and innovative financing mechanisms. These findings inform guidance for health systems strengthening amidst accelerating climate change.

## INTRODUCTION

Over recent decades, health systems (HS) worldwide have confronted challenges that strain their capacity to respond to the demands required to secure health and deliver high-quality and equitable health services^1^. In Latin America and the Caribbean (LAC), HS have been under pressure for a long time as a result of a variety of factors, including fragmented governance; insufficient funding, staffing, and infrastructure; challenges with information systems, and varying social inequities. Political instability, competing policy priorities, and limited technological advancement exacerbate these difficulties, leaving HS with a constrained capacity to effectively respond to ongoing and emerging health threats, especially in remote and disadvantaged areas^2^.

In this context, climate change introduces an additional layer of complexity. Extreme weather events (e.g., hurricanes, heavy rainfall events, and floods) disrupt access to healthcare, impact supply chains, and damage critical infrastructure, while shifting precipitation patterns, droughts, and rising temperatures contribute to vector-, food-, and water-borne diseases and place new and additional burdens on service delivery^3^. In LAC, climate-sensitive health outcomes are already manifesting: population exposure to heatwaves and subsequent heat-related illnesses have increased over the last two decades^3^. Potential dengue transmission has expanded in most countries, with, for example, severe emerging cases in previously unaffected areas of São Paulo^4^. Additionally, sea level rise is posing challenges for coastal countries related to flooding, infrastructure loss, food insecurity, injuries, and population displacement^3,5^.

Complementarily, HS contribute to climate change as they are responsible for around 5% of annual greenhouse gas (GHG) emissions globally, with emissions in LAC ranging from 18.8 kg CO_2_e per capita in Paraguay to 624.5 kg CO_2_e per capita in Panama^6^.

The World Health Organisation and the Pan American Health Organisation (PAHO) have promoted the development of climate resilient and low carbon (CRLC) HS^7^. These systems are those *"capable of anticipating, responding to, coping with, recovering from, and adapting to climate-related shocks and stress, while minimizing GHG emissions and other negative environmental impacts to deliver quality care and protect the health and wellbeing of present and future generations"* ^7^ (p.2). Based on this, CRLC-HS actively build capacities that reduce vulnerabilities, ensure continuity of care, and promote long-term resilience in the face of climate- and climate change-related hazards. The shift towards lower carbon intensity involves adopting cleaner energy sources, enhancing energy, water, and efficiency of all processes, and implementing sustainable procurement and waste management practices. It also encourages systemic changes in how healthcare facilities are designed, operated, and governed.

Although there is a growing body of global evidence on this area, systematic regional and local evidence from LAC remain scarce^8^, limiting contextual and cultural understandings that influence further progress. In this sense, the objective of this study was to systematically explore the progress, challenges, enablers, and lessons learned for building CRLC-HS in LAC. The analysis combined results from the PAHO 2021/2022 and 2023/2024 Climate Change and Health survey and semi-structured interviews with national focal points and technical experts in four countries in LAC.

## METHODS

### Study Design and Framework

This is an explanatory, sequential, mixed-methods study that integrates complementary quantitative and qualitative approaches. The sequential design was selected because the quantitative survey data revealed patterns regarding progress requiring deeper exploration. The qualitative phase was necessary to understand the mechanisms, contextual factors, and stakeholder perspectives underlying these patterns, which survey data alone could not capture. As a first step, we quantitatively analysed the PAHO 2021/2022 and 2023/2024 Climate Change and Health surveys (PAHO surveys hereafter) at the country level, which provided details about the progress on CRLC-HS. Based on these results, we identified a set of countries and conducted interviews with representatives/experts form each to further explore the challenges, enablers, and lessons learned in relation to that progress. We followed the Strengthening the Reporting of Observational Studies in Epidemiology (STROBE)^9^ and Consolidated Criteria for Reporting Qualitative Research (COREQ)^10^ reporting guidelines. Supplementary information and tables are contained in the Appendix ^a^.

The PAHO climate change and health agenda that guided the overall analysis (Section A in Appendix), and which aligns with the WHO framework, comprised analyses of:

1. Governance and intersectoral action structures;
2. Planning and regulatory frameworks;
3. Health surveillance and integrated information systems;
4. Climate and health finance;
5. Primary care and health infrastructure;
6. Clean, healthy and sustainable environments;
7. Emergency preparedness and response, and
8. Research and capacity building.

### Participants and Data Collection

The geographical scope covered all 33 countries in LAC: Antigua and Barbuda, Argentina, Bahamas, Barbados, Belize, Brazil, Bolivia, Chile, Colombia, Costa Rica, Cuba, Dominica, Dominican Republic, El Salvador, Ecuador, Grenada, Guatemala, Guyana, Haiti, Honduras, Jamaica, Nicaragua, Mexico, Panama, Paraguay, Peru, Saint Kitts and Nevis, Saint Lucia, Saint Vincent and the Grenadines, Suriname, Trinidad and Tobago, Uruguay, and Venezuela.

We analysed PAHO surveys, which were led and managed by PAHO and answered by focal points at the Ministry of Health (MoH) of Member States in LAC, in consultation with representatives of other sectors. This survey collects standardised national-level information related to health system performance, preparedness, and policy implementation in the context of climate change. The 2021/2022 PAHO survey included 36 questions, and answers were collected throughout 2021 and 2022 years. The 2023/2024 PAHO survey included 30 questions, and answers were collected throughout 2023 and 2024 years. The reduction in questions between surveys reflected PAHO's internal refinements to avoid redundancy and improve clarity, which had no implications for this study. We present changes comparing the same questions for both time points or just the state for one time point when appropriate.

After the analyses of the surveys, we purposively selected countries based on the following criteria:

1. Countries with demonstrated progress in any of surveys’ components;
2. Representation across different LAC sub-regions;
3. Diversity in health system structures (federal vs. centralised); and
4. Willingness and availability of MoH focal points to participate.

A total of six country focal points were invited following PAHO's procedures and communication channels, with Argentina, Chile, Jamaica, and Peru finally participating in a 1-hour online semi-structured interview. Interview and procedural information were first explained via email. At the beginning of the interview, participants provided their verbal consent to participate and record the interview. All interviews were carried out in English or Spanish, between March and August 2025. The final number of four interviews was deemed appropriate for an exploratory phase focused on depth rather than breadth, and aligns with guidance for qualitative studies examining complex institutional processes^11^.

The interviews aimed to capture contextual insights, strategies employed, key decision-making processes, and enablers and challenges to the implementation of actions for building CRLC-HS. We prepared guiding questions in relation to specific components, allowing for further exploration depending on how the interview progressed (https://doi.org/10.17605/OSF.IO/QC7X8).

Each interview involved between three and four key informants at the MoH and PAHO Country Office Focal point, and three to four members of the research team. All personal information of participants was kept confidential and anonymised.

### Data Analysis

#### Quantitative data analysis

Country-level data from the PAHO 2021/2022 and 2023/2024 surveys were analysed and mapped in alignment to the PAHO agenda. Descriptive statistics (i.e., proportions and summary statistics) and comparative analyses between surveys were performed to identify differences and patterns between and across sub-regions: the Caribbean (Car), Central America (CA), and South America (SA). The denominator for all proportions considered the countries per sub-region (Car = 14; CA = 9; SA = 10). Statistical tests were not applied as this is a descriptive census-level study of nearly all LAC countries. All tables with specific details are presented in the Appendix Section D.

#### Qualitative data analysis

All interviews were audio-recorded and electronically transcribed verbatim in the original language. The qualitative analysis software Delve (https://delvetool.com) was used for coding, theme management, and maintaining an audit trail of analytical decisions. Using consensus coding and individual memos, two researchers (YPS, CLl) independently check the audio and read and analysed the scripts, identifying key information respective to the guiding questions (deductive coding) and emerging codes regarding challenges, enablers, and lessons learned throughout different processes of building CRLC-HS (inductive coding). Once emerging codes were identified, three researchers (YPS, CLl, ZV) analysed the codes and clustered them into themes, continuously discussing to resolve any discrepancies and ensure the final thematic structure was a true representation of the key informants’ experiences and perspectives. Discrepancies were resolved via consensus discussion; no formal inter-rater reliability coefficient was calculated given the exploratory nature of this phase.

Respondent validation was conducted with all participants between September and October 2025. Participants received a document containing the thematic structure and key quotes attributed to their context. They were given two weeks to review and provide feedback. The final thematic structure incorporated all feedback received.

#### Ethical Considerations

Ethical approval was obtained from the Universidad Peruana Cayetano Heredia prior to data collection (SIDISI N°216873/ CIEI-73-8-25). Informed consent was obtained from all interviewees, and confidentiality measures were strictly adhered to. Data were de-identified, securely stored, and accessed only by authorised members of the research team to ensure the privacy and protection of all participants.

## RESULTS

Of the 33 countries in LAC, 24 (73%) participated in the 2021/2022 and 27 (82%) in the 2023/2024 surveys, with four common non-participating countries in both (Section C in Appendix). Interview participants included three people from Argentina, four people from Chile, three people from Jamaica, and three from Peru.

### Key Findings by Component and Area

Overall, the data reveal varying levels of progress across the components of the PAHO agenda for CRLC-HS in the Caribbean, CA, and SA. Figure summarises the progress by sub-region.

**Figure:**
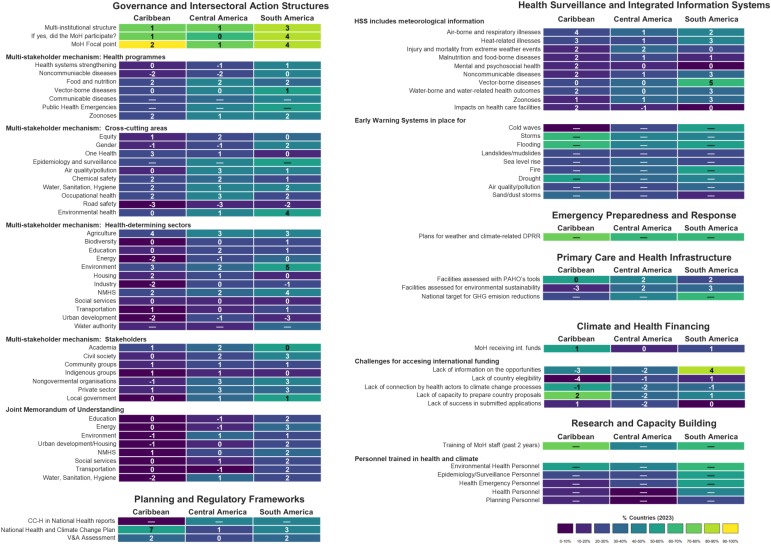
Climate and health indicators by sub-region.

Note: Colour intensity indicates the proportion of countries reporting "yes" to each indicator in 2023/2024. Numbers within cells represent the net change in the number of countries responding "yes" from 2021/2022 to 2023/2024 surveys.

#### Governance and intersectoral action structures

Across LAC, progress has occurred in establishing multi-institutional structures (e.g., national inter-ministerial committee, national coordination mechanism) for climate change. Car showed the strongest baseline, with ten (71.4%) countries having established structures in 2021/2022, increasing to 11 (78.6%) in 2023/2024. CA had a similar growth (six [66.7%] to seven [77.8%] countries), whilst SA went from five (50%) to eight (80%) countries. The MoH's participation in these multi-institutional structures showed similar findings, with Car showing high engagement (10 [71.4%] to 11 [78.6%] countries), and SA doubling from four (40%) to eight (80%) countries between surveys. CA remained at six (66.7%) countries both years. Focal point designation for health and climate change also showed progress, reaching 13 (92.9%) countries in Car, seven (77.8%) countries in CA, and eight (80%) in SA in 2023/2024.

However, multi-stakeholder mechanisms (e.g., task force or committee) within MoHs remain limited. Vector-borne disease and public health emergencies programmes showed the highest engagement (28.6%–50.0% of countries across regions), whilst noncommunicable diseases and health systems strengthening programmes showed the lowest (14.3%–40.0% of countries) in 2023/2024. Cross-cutting areas showed slight improvements between, with equity, One Health, chemical safety, Water, Sanitation, and Hygiene (WASH), occupational health, and environmental health as the areas with the highest improvements in up to 50% of countries.

Regarding the involvement of health-determining sectors/ministries (e.g., agriculture, environment, housing, industry) participating in the multi-stakeholder mechanism, the results were mixed. Agriculture showed one of the most notable increases, particularly in Car (from zero countries to four [28.6%]), and modest growth in CA and SA (adding two or three countries). Similarly, national meteorological and hydrological services had an important increase of about 2–4 countries across sub-regions. In contrast, some sectors, such as energy and urban development, showed a slight decrease in the number of countries reporting collaboration.

In terms of stakeholders and/or experts that participate in the multi-stakeholder mechanism, there were increases, with civil society, community groups, non-governmental organisations (NGOs), and the private sector being reported by more countries.

The reported establishment of formal memorandum of understandings (MoU) between MoHs and other health-determining sectors remained low across all sub-regions in both years. One (7.1%) country in Car, three (33.3%) in CA, and two (20%) in SA reported having MoUs between MoH and the Ministry or sector of the environment in 2023/2024.

#### Planning and regulatory frameworks

Inclusion of climate change into national health reports showed mixed results. By 2023/2024, four (44.4%) countries in CA and four (40%) in SA had included climate considerations, whilst only one (7.1%) in Car had done so. The development of national health and climate change plans or strategies showed slight advancement, with Car leading (seven countries [50%] having plans by 2023/2024, up from 0% in 2021/2022), followed by SA (n = 4 [40%] from n = 1 [10%]) and CA (n = 2 [22.2%] from n = 1 [11.1%]).

Vulnerability and adaptation (V&A) assessments showed moderate progress. By 2023/2024, five (35.5%) Caribbean countries reported having completed assessments (up from three [21.4%]), whilst two (22.2%) of CA and three (30%) of SA countries had done so. From those having V&A assessments, these mostly cover the national level, and the population groups most commonly considered are people over 65 years of age and women. No more than five countries in the region reported using V&A assessment findings to inform the development of the national health and climate change plan/strategy, policies/programmes, or resource allocation. Budget estimation for implementation remained critically low across all regions, with only one country in the Caribbean, one in CA, and two in SA having estimated required resources by 2023/2024.

#### Health surveillance and integrated information systems

While some progress was made in integrating meteorological information into health surveillance systems, overall coverage remains limited. By 2023/2024, six (60%) SA countries reported integrating meteorological information into vector-borne diseases surveillance compared to three in Car (21.4%) and CA (33.3%). This integration for heat-related illnesses surveillance also increased in Car (0 to 21.4%, n = 3) and SA (10%, n = 1 to 40%, n = 4). However, for many outcomes, such as impacts on healthcare facilities and mental and psychosocial health, there is still minimal integration of meteorological information across all regions.

By 2023/2024, early warning systems (EWS) for storms and flooding were widely reported in Car (64.3%, n = 9 for both), and to a lesser extent in CA and SA (four countries in both CA and SA reported EWS for storms, while four and five countries reported EWS for flooding). SA had a higher number of countries using EWS for forest fires and cold waves (50.0%, n = 5). Meanwhile, drought alerts were more prevalent in Car (57.1%, n = 8). Less than 30% of countries in each subregion reported having EWS for landslides/mudslides, sea level rise, air quality, and sand/dust storms.

#### Climate and health financing

Access to international funds remains a challenge for the majority of the countries, particularly in SA and CA. While half of Car countries reported receiving international funds (42.9%, n = 6 by 2021/2022 to 50%, n = 7 by 2023/2024), SA countries reported few recipients and minimal change (10%, n = 1 to 20%, n = 2), and CA countries remained the same at 11.1% (n = 1).

The primary reported challenges limiting access to international funds were lack of capacity to prepare country proposals (64.3%, n = 9 to 78.6%, n = 11 in Car) and lack of information on opportunities (40%, n = 4 to 80%, n = 8 in SA). Lack of country eligibility, which by 2023/2024 was no longer reported as a barrier in Car (28.6%, n = 4 in 2021/2022 to 0% in 2023/2024), remained a challenge for some CA (22.2%, n = 2) and SA (10%, n = 1) countries. The lack of connection by health actors to climate change processes slightly decreased, but still is a challenge (seven in Car, and three in CA and SA, each).

#### Primary care and health infrastructure

Assessments of health facilities for climate resilience showed some progress, but environmental sustainability assessments were less common. The proportion of countries reporting the country's public health care facilities have been assessed according to PAHO's Hospital Safety Index and Green Checklist for climate resilience increased in CA (22.2%, n = 2 to 44.4%, n = 4) and SA (0% to 20%, n = 2), but remained stable in Car (50% [n = 7]). In contrast, the proportion of countries reporting assessments for environmental sustainability in public healthcare facilities decreased in Car (35.7% [n = 5] to 14.3% [n = 2]) and only increased slightly in CA (11.1% [n = 1] to 33.3% [n = 3]) and SA (0% to 30% [n = 3]). Few countries reported national targets or recommendations for GHG emission reductions in the health sector by 2023/2024 (21.4% [n = 3] in Car, 44.4% [n = 4] in CA, 60% [n = 6] in SA).

#### Clean, healthy and sustainable environments

The use of tools to assess health co-benefits of climate change action remained limited. Only Cuba and Nicaragua in CA, and Argentina and Colombia in SA reported using tools such as AirQ+ or CLIMAQ-H (formerly CarbonH). No Caribbean countries reported using co-benefit assessment tools.

#### Emergency preparedness and response

By 2023/2024, more than half the countries had developed plans for weather and climate-related disaster preparedness, response, and recovery: ten (74.4%) in the Caribbean, six (66.7%) in CA, and six (60%) in SA. However, implementation of public health communication campaigns on climate change remained limited across all regions: five (34.7%) countries in Car, four (44.4%) in CA, and three (30%) in SA.

#### Research and capacity building

By 2023/2024, ten (71.4%) Car countries reported training MoH staff on climate change and health, followed by six (60%) in SA and four (44.4%) in CA. For specific personnel categories, environmental health personnel were most frequently trained. Training for other categories, such as health personnel (e.g., doctors, nurses) and planning personnel, remained low.

### Qualitative Insights on Implementation Barriers and Enablers

Participants from Argentina, Chile, Jamaica, and Peru shared their experiences related to the implementation of diverse actions for building CRLC-HS. Specific country contexts are provided in Boxes 1–4^12-22^.

Box 1 **Case and experience from Argentina**

In 2008, the MoH of Argentina carried out a national profile on climate change and health, which served as a basis for a situation analysis of health and climate change^12^ and the National Action Plan on Climate Change and Health^13^. These processes were supported by PAHO and a working group ("*Mesa de Trabajo de Cambio Climático y Salud*") that convened several departments within the MoH, including people from other agencies at the provincial level. Based on this work, the readiness proposal "*Increasing health sector's capacities and strengthening coordination on climate action in Argentina at national and subnational levels*" was submitted to the Green Climate Fund (GCF) and granted, allowing for actions towards increasing the coordination across government levels on this topic^14^. The provinces of Misiones (Northeast region), Tucumán (Northwest region), and Neuquén (Patagonia region) were selected to represent different climate and social characteristics. Main activities included: identifying capacity gaps and needs, preparing actions plans, measuring carbon emissions of healthcare facilities, estimating health co-benefits and economic costs for different emission pathways proposed in the National Determined Contribution (NDC), proposing a national climate and health data integration platform, and developing communication and outreach strategies^14^. The implementation involved extensive stakeholder engagement, including a consultation and participatory workshops bringing together 19 ministerial areas, indigenous peoples, and youth organisations. The project successfully established working groups at the national and provincial levels, with participating provinces now supporting neighbouring regions in developing their own health and climate change plans.

In Neuquén province, a Provincial Working Group on Health and Climate Change (MeSaCC) was established to mirror and coordinate with the national group, facilitating the integration of climate considerations into provincial health policies and programs. The process included the development of Terms of Reference and the drafting of the 2023 Provincial Action Plan on Health and Climate Change^15^, spanning up to 2030 and structured around four pillars: governance and intersectoral coordination; evidence generation and epidemiological surveillance; sustainable and climate-resilient health infrastructure and services; and communication and capacity building. Pilot activities included the measurement of carbon footprints in health facilities and participatory workshops with stakeholders from the six provincial health zones, technical units of the MoH, and municipalities. This subnational experience highlights the role of provinces as laboratories for adaptation and innovation, generating lessons to inform national strategies.

Box 2 **Case and experience from Chile.**

Chile has been characterised by having robust and longstanding systems for surveillance and management of environmental health determinants, including air pollution, water quality, and waste management^16^, which are core components of national environmental and health policies. For climate change and health, there has been a growing prioritisation of climate change across all policies over the last decade, with the Climate Change Framework Law Nº 21455 and the inter-ministerial committee being relevant drivers and enablers of this process. Chile approved the first Health National Adaptation Plan (HNAP) in 2017 and developed the second HNAP during 2023/2024, alongside the first Health National Mitigation Plan, integrating several lessons from previous versions. Additionally, the current National Health Strategy to 2030 explicitly included climate change as an element of the strategic component of "emergencies and disasters"^17^. A significant strength for this progress is a close collaboration among diverse departments within the MoH and the strong leadership of the team in charge of climate change and health plans. Recently, the MoH Department of Emergency and Disaster Risk Management, which hosts climate change and health plans, was elevated to a Division (3^rd^ level top-down) with climate change positioned within the Preparedness Department. This structural integration aims to ensure that climate considerations inform preparedness and response to climate change-related events such as the re-emergence of *Aedes aegypti* in continental Chile. Additionally, this new division has been an active part of the multi-sectoral working group leading disaster risk management plans for extreme temperatures at different governmental levels.

Box 3 **Case and experience from Jamaica.**

Jamaica has been one of the countries in the region that has made significant progress in producing V&A assessments at the subnational level, supported by PAHO/GCF readiness projects and national development initiatives. It has been highlighted that the conducive environment for this work stems from the National Development Plan Vision 2030, which is the country's first long-term strategic development plan from 2009 to 2030 and includes climate change as a priority^18^, and the establishment of the Climate Change Division, which aims to facilitate climate action across sectors in the country through the focal point network. Health has been prioritised as a key sector, allowing for the planning and implementation of a diverse range of activities related to climate change and health. In addition to the policy and governance structure, collaboration with other sectors and institutions has facilitated this progress. The University of the West Indies (UWI) Climate Studies Group in Jamaica and the Meteorological Service of Jamaica have been fundamental, allowing the development of *The State of the Jamaican Climate Report* ^19^ and *The State of the Caribbean Climate Report* ^20^. All these efforts provided the groundwork for further high-level actions in the health sector, including the Smart Project^21^, infrastructure assessments, sensitisation and awareness sessions for the healthcare workers and other key actors, and the first Caribbean Action plan. A more recent initiative is the Climate Change and Health Leaders Fellowship, which trains individuals to integrate climate issues into their work and foster cross-sectoral partnerships, further supporting collaborative work on climate change and health.

An important element across all these processes and initiatives is that Jamaica's approach leveraged existing and concrete issues related to climate, climate change, and health. The country strategically built upon existing concerns around infrastructure resilience, particularly hurricane preparedness, to introduce broader climate-health concepts. For example, this approach enabled the integration of new dimensions as capacity developed, including air quality monitoring and medical solid waste improvements. These progressive and concrete links facilitated building awareness, stakeholder engagement, cross-sectoral action, and sustained progress.

Box 4 **Case and experience from Peru.**

Peru has prioritised strengthening its human resources on climate change topics, developing training programmes for MoH (*Ministerio de Salud* — MINSA) staff at national and regional levels. The Ministry's initial attempts at climate change planning began in 2014, after the country hosted COP20. Subsequently, MINSA contributed to the elaboration of Peru's Nationally Determined Contributions between 2016 and 2018. However, a dedicated office to lead a more formal process on climate change and health was established in late 2020 within the General Directorate of Disaster Risk Management and National Defense in Health (DIGERD).

A critical first step was institutionalising this commitment through a ministerial resolution. This resolution designated DIGERD as the focal point for climate change and health and established a permanent working group ("*Grupo de Trabajo Sectorial para la Gestión Integral del Cambio Climático del Ministerio de Salud*") composed of more than 18 MINSA general directorates^22^. Peru has focused on regional engagement, ensuring all regions have their own working groups and long-term plans (up to 2030 and 2035) that feed into national goals. The MINSA initially identified an ambitious plan of 11 outputs and 14 adaptation measures, including technologies for the improvement of health infrastructure, strengthening capacities at different governmental levels, access to health finance, and strengthening of monitoring and surveillance systems. However, due to resource and capacity constraints, these were re-organised and prioritised.

Formal training was a central component of this strategy, as it was understood that progress would be limited without a well-trained workforce. Peru's first professional training programme was highly ambitious. Its difficulty level contributed to low approval rates, necessitating reassessment for future versions to include more geographically relevant and intercultural learning materials. Additionally, the need for sustained funding and enhanced awareness remains critical to advancing the ministry's work on climate change and health.

#### Challenges and barriers

Progress towards CRLC-HS in the region faces several entrenched challenges, often rooted in awareness, capacity, and resource constraints. An important factor is the perception of climate change as a distant and abstract problem, which seems to be associated with two main challenges: overload and competing factors, as well as resistance.

Decision-makers and key actors are often "overloaded" with tasks and have "multiple roles", making it difficult to absorb new tasks related to climate action. Because of this, they often prioritise daily and immediate pressures over long-term climate planning and programming. This affects the planning, implementation, and even financial sustainability of actions towards adaptation and mitigation in the sector.

*"…even though we know that all these ten things need to be done, we can only do two or three, not because the others are not important, but you will have to do a prioritization and to see what's next."* (Interviewee ID6)

*"The problem with climate change is that we have always addressed it as a long-term issue, meaning 2030, 2050, and no authority or professional or staff member of the ministry is concerned about what will happen in ten or 20 years. Everyone is concerned about what is happening right now"* (Interviewee ID3)

There seems to be a degree of resistance among decision-makers regarding a perceived lack of mandate (i.e., feeling they lack the institutional authority to act). This is compounded by little acknowledgement of how the issue links to their day-to-day activities and responsibilities, which is mostly driven by limited awareness and knowledge.. However, this barrier has been identified as "overcomeable" by sensitisation processes.

*"…it was very difficult to generate involvement in the issue, something they believed they had nothing to do with"* (Interviewee ID2)

*"The first thing we did was an awareness-raising activity within the Ministry of Health. That's where resistance to the issue arose. All areas were involved, from hospital infrastructure, maternity, to senior care. Some were perhaps more easily connected to the topic, but others didn't know why they were there at all. So, [during] this reflective activity, they made that connection between topics, areas, and evidence, and said, ‘Oh, look, it actually is [my concern], now that I think about it.’ These same representatives, who later became part of the health and climate working group, will serve as facilitators in [further] consultation workshops"* (Interviewee ID2)

Financing was identified as a barrier; however, differences exist. In some countries, climate plans and activities were often assigned without corresponding budgets, forcing reliance on external partners and triggering sustainability concerns. Other countries, classified as "high-income countries" based on Gross Domestic Product, face the challenge of being non-eligible from some international aid, limiting access to climate finance available to other LAC countries and pushing such countries to rely almost entirely on national budget and other strategies.

*"Unlike other countries in the region, we depend heavily on the national budget. This has necessitated strategic resource management, identifying actions that are achievable through reorganisation rather than new funding, whilst pursuing alternative sources like NDC [National Determined Contribution] funds for specific components such as training"* (Interviewee ID8)

A significant challenge emerged around differential needs and knowledge levels across governance tiers and lack of tailored training, creating important implementation barriers. Although training programmes on the topic are available, they often fail to address local contexts or use terminology compatible with national regulations, affecting their impact. Even some courses created on the topic and delivered by national organisations did not achieve their learning outcomes due to persistent gaps between what was taught, what was locally required, and the level of basal understanding of the topic.

*"There are actually many courses from institutions, and they're interesting, they're good, they complement the work, but it's a very different thing when you talk to your colleague and [they] say, ‘Hey, look in our office, in our ministry, this is our reality, and this is how we have to approach it, it's totally different’".* (Interviewee ID3)

A shared challenge across countries was siloed operations and communication barriers, adding inefficiencies along the processes and hindering progress.

*"…having the capacity to process all that information, I think that's what seems complex. At the regional level they also have to make plans and communal plans, so, they're also analysing hazards at the [local] level and transmitting the information to us [central level]. Integrating the information is not easy."* (Interviewee ID4)

Other elements that contribute to the communication challenges are linked to the diverse use of technical jargon and limited efforts to have a common understanding and language across sectors. This is exacerbated by frequent personnel changes linked to political cycles, disrupting continuity and institutional memory.

*"…sometimes the language or the understanding becomes a barrier in and of itself. Because we speak, we tend to provide our discourses along the jargons and lines that we are comfortable with in our respective technical areas. So, there are often times when we've had to engage disciplines. Most persons will nod and say yes, and they get that. Translating that into action is an issue, unless they can specifically identify it in advance."* (Interviewee ID6)

#### Opportunities and enablers

Despite the significant challenges, various opportunities and enablers exist for advancing CRLC-HS in the region. A key opportunity lies in building upon existing efforts and leveraging pre-existing awareness. Associating climate action with established health priorities or prevalent climate-sensitive diseases or outcomes, such as vector-borne disease control or disaster preparedness, makes the topic more tangible and demonstrates clear co-benefits.

*"…after we finished the Smart Project, we moved into air quality considerations at health facilities. I know we're having the conversation around air quality in general as a mitigation focus. So right now the structure in place is to tap into the energy sector, and then there are the other elements that need to speak to it. We would have established a mechanism for information exchange, and so by adding on the additional dimensions in relation to what needs to happen to push through on the survey and the HNAP."* (Interviewee ID6)

*"So, if we start talking about health co-benefits, it's information that resonates [among decision-makers]. We have to go with the most accurate and condensed information that has an impact, [and say] ‘So, look at how many fewer accidents and heart attacks you'll treat if we improve this factor?"* (Interviewee ID1)

A related enabler is the role of evidence to inform processes and decision-making. Evidence and data coming from different sources, especially scientific evidence on the status and progress of climate change-related hazards, guide activities, their prioritisation, and help show the relevance of the topic.

*"Among other things, we're taking advantage of evidence of climate change, such as the advance of [Aedes] aegypti. So, there's no one who wants to deny that climate change exists…"* (Interviewee ID1)

From a more systemic perspective, strong governance, robust institutions, and supporting legal regulations are fundamental enablers for sustained action. Establishing dedicated units, focal points, and permanent working groups provides a robust institutional foundation that ensures work continuity, giving a long-term perspective, independent of changes in people or decision-makers.

*"So, what we did first was put our house in order. We had the ministerial resolution, which in reality it is something that might sound a bit administrative and bureaucratic to you. But we got off to a very good start with a ministerial resolution designating the directorate as the focal point for climate change. And then we began to put together a climate change working group with more than 18 directorates-general, with the Technical Secretariat, or the coordinator, as the focal point."* (Interviewee ID3)

As part of a strong governance, multi-sectoral and inter-ministerial committees greatly facilitate coordination and the integration of health priorities into other sector plans, including the integration of climate change into long-term national planning and at different governance levels.

*"[The Ministry's] approach also focuses on emergencies, but there is also internal coordination between the environmental and emergency teams to address these issues, where information is shared. Furthermore, the Ministry's team has been working on multi-hazard plans, which incorporate climate change at national and regional levels."* (Interviewee ID5)

Establishing direct and clear communication lines between departments and sectors, and fostering alliances with external entities, such as universities and meteorological offices, strengthens discourse, promotes collaboration, and provides essential data.

*"…we realised that most of the persons involved had multiple roles. So we had to make sure that we do all the groundwork. We are specific. We are objective in our meetings so that persons don't see it as another task that is mundane. So that is important, and keeping the communication going outside of the meeting time. So then, when we come to meet, we are looking at the issues and the actions to be undertaken."* (Interviewee ID7)

*"[As a public entity] our responsibility is so high that we should be very well-equipped, meaning the staff should know how to manage [engagement]. You, the allies, may have the best intentions, want to do a lot, but if you don't work hand in hand with the public entity, which is the one that ultimately executes and dictates the rules, we lose many opportunities there."* (Interviewee ID3)

Multi-stakeholder engagement that brings together government, academia, civil society, and even indigenous groups creates several synergies and learning opportunities. Furthermore, external partnerships and technical support from international organisations are crucial for enabling progress, especially in countries with limited national budgets. However, all this requires diplomacy as a critical element for better mutual understanding and effective collaboration.

"…*it really demands a very strong trait in diplomacy to allow for the amalgamation of the different forces, so that motion actually happens, right? Because otherwise we have a full day workshop, and everybody goes in their respective directions. Another country doesn't move forward."* (Interviewee ID6)

Complementarily, decentralised or federal structures offer an opportunity for subnational governments to pursue climate initiatives autonomously, creating opportunities for context specific implementation and models for scaling-up, and ensuring continuity even when national policies face scepticism.

The presence of key individuals, particularly within MoHs, with strategic vision is another critical enabler. These committed individuals who recognised the opportunity of addressing climate change and health can drive initiatives and bridge traditional siloed areas and/or strengthen further collaboration. Their personal drive and ability to show policy- and action-relevant information can be pivotal in gaining buy-in and promoting further dialogue.

#### Lessons learned

The experiences of countries in LAC emphasise the need for strategic, adaptive, and collaborative approaches. A primary lesson is the critical importance of institutional anchoring. Formalising climate responsibilities through official resolutions and embedding them into key institutional documents ensures continuity independent of political cycles. This "institutionalisation" proved more sustainable than relying on individual champions alone.

Another key lesson is the value of creating safe and participatory spaces for learning and discussion. Meetings and workshops in a non-judgmental environment allow individuals to express what they think and can be excellent spaces where initial resistance can be addressed. Then, intentional and structured activities, tailored to the audience's needs, encourage participants to make their own connections between topics and evidence, leading to a profound appropriation of the thematic.

*"…[it is important to empower] those who are against it to speak up, to express their opinions, to kick up a fuss if they want to — but I think that's what most moves people from their safe spaces."* (Interviewee ID2)

*"The resources [from previous engagement activities] were utilised to facilitate those consultations and then build on it, put it into our local context. So, when we engage, for example, with transport, we have to be very dynamic in how we have the conversation. And so there's a core set of information that is standard, and everybody gets. But we now have to tailor it to our respective audiences."* (Interviewee ID6)

Including diverse groups, such as indigenous peoples and youth organisations, has also led to transformative learning experiences for all participants.

*"I learned things I never would have encountered if not for the opportunity to convene around climate change"* (Interviewee ID1)

The experiences highlight the need for tailored capacity building. Effective training can initiate at the MoH at different levels and also account for varying baseline knowledge and local realities. One key approach is training "integral managers" or "knowledge brokers" rather than relying solely on deep technical specialists. These individuals, equipped with both technical expertise and strong diplomacy skills, can identify opportunities, coordinate across sectors, and drive effective implementation, bridging critical gaps between national and local levels.

*"We need to strengthen the workforce. We can have everything, but if people don't understand climate change, for example, how to apply for a project or a fund, we're stuck… I mean, there are regions that do have a budget, but what they lack is the management itself. I mean, they have allies but don't know how to do it, so they lack those professionals or those leaders who can guide the work and say, ‘Hey, we need to partner with this company, this NGO, this university…’"* (Interviewee ID3)

Strategic thinking, persistence, and "ant work" (i.e., meticulous hard work) are essential. Integrating climate change into health requires continuous effort to identify opportunities for integrate the topic across various contexts and link it to everyday issues.

*"…wherever there was a gap, you have to get the topic in, I think, little by little. It's ‘ant work’".* (Interviewee ID1)

Lastly, given resource constraints and differential capacities within countries, prioritisation and realistic ambition are critical to ensure that plans are executable, achievable, and have the intended impact.

## DISCUSSION

This study is one of the first systematically examining progress, challenges, and opportunities for building CRLC-HS in LAC. We integrated quantitative survey data from almost all LAC countries with in-depth insights from four of them. The results confirm that, while the foundations for CRLC-HS are being established, progress is uneven, and the transition from planning to effective implementation is constrained by a complex interplay of factors spanning technical capacity, institutional silos and fragmentation, resource scarcity, and political economy dynamics.

Overall, notable progress in governance structures has happened in LAC between 2021/2022 and 2023/2024, with the majority of countries reporting the establishment of climate change focal points and multi-institutional mechanisms. However, there seems to exist a persistent gap between establishing administrative formal structures (e.g., focal points, committees) and achieving substantive integration and implementation (e.g., programme modification and intersectoral agreements). Fewer than half of countries have integrated climate change into national health reports by 2023/2024, and amongst those few completing V&A assessments (less than 40% of countries), minimal use of findings to inform policy development, strategic planning, or resource allocation was reported. This situation creates an important gap between the use of V&A or climate risk assessments and policy development. Plans or programmes that do not include the role of climate and climate change might fail in achieving their goals as weather patterns are rapidly changing, affecting population health, and will continue to do so.

Qualitative analysis reveals that this gap might stem from systemic issues: climate change and health is still perceived as abstract and distant to health and non-health sectors, and competes with more "immediate" health issues for capacity and resources. Financial constraints are universal, with countries either lacking dedicated budgets or being ineligible for international climate finance. A key challenge is the limited capacity of the workforce, as knowledge asymmetries across diverse governance levels and persistent sectoral silos further impede progress. Also, the limited uptake of V&A assessments into policy and resource allocation reflects fundamental disconnects between technical and decision-making processes, which is even more critical when assessments focus on national-level analyses, potentially limiting the relevance for sub-national decision-making where resource allocation and implementation often occur.

This gap and findings suggest that creating committees and assigning focal points might require minimal additional investment, and generate short-term political visibility and a potential sense of accountability. While helpful, this investment might not fully translate into climate-informed health programmes and operational actions, which may require more complex efforts, additional collaboration lines, sustained funding, technical capacity, and strong political commitment.

Additionally, our findings highlight that community engagement in designing and implementing actions for CRLC-HS remains underdeveloped, with community groups and indigenous peoples minimally represented in multi-stakeholder mechanisms and inconsistently considered in V&A assessments. These mechanisms might be associated with contextually inappropriate interventions that fail to address community-prioritised needs or leverage local knowledge. Argentina and Jamaica's experiences suggest participatory approaches (e.g., consultation workshops, indigenous peoples’ inclusion, subnational engagement) strengthen both appropriateness and ownership of climate-health responses. Moving forward, community participation can be integrated beyond general consultation to co-design of adaptation strategies, requiring sustained investment in participatory methodologies, culturally appropriate communication strategies, and governance structures^23^.

A final example of the plan-implementation gap is represented by the existing divergence between weather and climate-related disaster preparedness plans and implementation of public health communication campaigns, reflecting differential capacity requirements: this planning leverages long-existing technical disaster management expertise and core health sector services^24^, whilst sustained communication campaigns demand specialised health communication skills, ongoing operational budgets, and complex multi-channel strategies for diverse populations. Once again, emergency response generates immediate political visibility, whereas prevention-focused communication delivers diffuse and long-term impacts extending beyond political cycles. This gap highlights the importance of adjusting and reinforcing activities towards informing and educating people, which is a public health function, on the health risks of climate change.

Taking all this into account, as well as international evidence, a critical gap emerges from this analysis: the near-absence of systematic evaluation frameworks and activities for assessing the implementation and effectiveness of climate change, climate, and health policies and programmes^25^. Whilst the region has made progress establishing governance structures and developing plans, mechanisms to evaluate whether these interventions are implemented and achieve intended outcomes (e.g., reduced climate-attributable morbidity, health service continuity during extreme events, or decreased health sector emissions)^26^ remain critically underdeveloped. This monitoring and evaluation deficit is problematic in a changing climate, where the effectiveness of current and potential interventions may diminish as population exposures intensify or shift geographically.

To address this concern, establishing and implementing robust monitoring and evaluation (M&E) processes from programme inception, rather than retrospectively, would contribute to effective health adaptation and evidence-informed resource allocation. Such frameworks should capture information and indicators including health outcomes, health systems dynamics, intermediate process measures, and generate locally relevant evidence of "what works" in LAC contexts. Administrative governance structures and siloed plans and programmes without systematic M&E contribute to perpetuating ineffective approaches or missing opportunities to scale interventions, undermining investments already made in planning and capacity building.

When compared globally, HS in LAC shows mixed progress. European countries have advanced further in implementing climate-health strategies, with the UK's National Health Service (NHS) committing to net-zero emissions by 2040 and establishing dedicated sustainability units across trusts^27^. Asian nations and cities have developed heat-health warning systems, observing benefits in health outcomes^28^. African countries, whilst facing similar resource constraints to LAC, have leveraged international partnerships more effectively, with the "Climate Change and Health: Strategic Framework 2025" being led and published by the AfricaCDC^29^. In this sense, LAC's progress appears to be intermediate: more advanced than low-income settings in establishing governance structures, yet lagging in implementation and M&E compared to high-income regions and some middle-income countries that have prioritised climate-health integration.

Considering all of the above, the need for robust CRLC-HS in LAC seems to be particularly relevant given the additional region's exposure to climate hazards, vulnerabilities among populations^30^, contribution to GHG emissions^6^, other pollutants, and waste. However, multiple overlapping challenges arise.

First, LAC countries have to deliver health services and manage climate-sensitive disease burdens that are changing their usual patterns^30^, which clashes with mixed and rapidly changing populations in terms of epidemiological and demographic profiles^2^. Storms and hurricanes in Car and CA, vector-borne disease expansion in SA, and extreme weather events across the region are adding unprecedented pressure to the already overwhelmed HS in LAC. Therefore, HS need to prepare and plan for anticipating and responding to new health demand patterns by considering climate change-related hazards as determinants of health.

Second, added to the new climate change-driven health patterns, HS also have to deal with historical health inequities. LAC is one of the most socially unequal regions in the world, not only in terms of monetary income but also in terms of access and opportunity to health services, from promotion and prevention to treatment^2^. This situation limits effective adaptation, and climate change creates vicious circles between exacerbation of inequities and poor health.

Finally, although HS in LAC are not big GHG emitters compared to other countries in the world, they still contribute to these emissions, other local pollutants, and waste. Therefore, mitigation and adaptation actions can (and should) go hand-in-hand, with many interventions bringing local co-benefits for health and proving cost-saving in their own right. For example, solar panels reduce GHG emissions and increase grid independence during potential disasters, whilst green building designs generate operational savings through improved energy efficiency^31^.

Our findings emphasise that whilst technical measures are essential (e.g., V&A assessments, emission inventories, and resilient infrastructure standards), these alone are insufficient. Countries’ insights demonstrate that progress depends equally on "soft" and political factors: strategic partnerships, sustained political commitment, diplomatic skills to navigate multi-sectoral dynamics, and investment in knowledge and capacity.

Based on our findings, HS in LAC can:

1. Go beyond traditional metrics of tracking disease incidence and climate hazards by working on and integrating national climate-health observatories or centres as a potential mechanism for monitoring process and implementation indicators reflecting institutional maturation;
2. Assessing the operational status of multi-stakeholder mechanisms and sectoral representation;
3. Supporting formal inter-ministerial agreements;
4. Training dedicated climate-health personnel and their continuity despite political cycles;
5. Analysing budget allocation and financial flow trends;
6. Tracking utilisation rates of V&A assessments in policy documents; and
7. Surveying training coverage across diverse health workforce categories.

External partnerships with, for example, PAHO, development banks, and academic institutions proved crucial for all countries, providing not just funding but technical expertise, continuous support, and legitimacy. Boundary organisations in LAC might support countries in implementing actions and measures by bridging scientific, policy, and practice communities^32^.

This study has several limitations. First, survey responses are self-reported, which might add some reporting biases, potentially leading to overestimation of progress and hiding details in terms of actual implementation levels across countries. Second, survey non-responders may represent countries with fewer initiatives revolving around climate action, which might overestimate progress happening in the region. Countries with stronger climate-health governance may be more likely to participate in surveys, meaning our findings may not reflect the full range of regional challenges. Third, the survey captures reported actions rather than implementation quality or health outcomes. Fourth, the rapidly evolving nature of climate-health initiatives means our snapshot in 2023/2024 may not reflect recent developments, especially as countries prepare their new NDCs. Fifth, interviews were conducted with only four countries, limiting generalisability across LAC's diverse contexts and misrepresenting barriers faced by countries with stagnant or declining progress. However, the qualitative data provided crucial context and revealed that these countries share similar fundamental challenges related to a lack of funding, limited workforce capacity, and a tendency to work in institutional silos.

It might be desirable that future research employ longitudinal designs, include systematic and structured implementation frameworks, such as RE-AIM or PRISM frameworks to guide the planning and evaluation of programmes, track implementation outcomes, expand qualitative sampling to include more countries and subnational perspectives, and develop standardised metrics for assessing health system climate resilience and low carbon development.

Moving forward, LAC countries require differentiated support recognising varying capacities and contexts. For example, small island developing states might need targeted assistance for climate-resilient infrastructure and population relocation, whilst larger countries may benefit from technical support for low carbon development and mitigation strategies. Regional platforms for knowledge exchange and collaboration, exemplified by the Caribbean Action Plan, could support learning and accelerate action across similar contexts. Finally, and critically, climate-health integration must be framed and seen not as an additional siloed task within the health sector, but as an embedded essential lens for achieving CRLC-HS that is capable of protecting population health amidst accelerating climate change.

## Data Availability

The raw data is available upon request from the corresponding author.

## Footnotes

a Available from: https://doi.org/10.17605/OSF.IO/QC7X8
