# Supplementary material for: Progress, challenges, and lessons for climate resilient and low carbon health systemsin Latin America and the Caribbean: an exploratory mixed-methods study
Source: Rev Saude Publica. 2026 Jul 6;60:e24. doi: 10.11606/s1518-8787.2026060007430 (PMC13336743; doi:10.11606/s1518-8787.2026060007430)
Supplement: Appendix [file 1518-8787-rsp-60-e24-Suppl01.pdf]

Appendix

Progress, Challenges, and Lessons for Climate Resilient and Low Carbon Health Systems in Latin America and the Caribbean: An exploratory mixed-methods study

Yasna Palmeiro-Silva. Center for Health and the Global Environment, University of Washington, Seattle, WA, USA. Institute for Global Health, University College London, London, UK. palmeiro@uw.edu

Camila Llerena-Cayo. Centro Latino Americano de Excelencia en Cambio Climático y Salud, Universidad Peruana Cayetano Heredia, Lima, Peru. CAMILA.LLERENA.C@upch.pe

Sebastian Bauhoff. Inter-American Development Bank, Washington, DC, USA. sbauhoff@iadb.org

Kris L. Ebi. Center for Health and the Global Environment, University of Washington, Seattle, WA, USA. krisebi@uw.edu

Catharina Giudice. Department of Emergency Medicine, Boston Medical Center, Boston University School of Medicine, Boston, MA, USA. catharina.giudice@gmail.com

Cristian A. Herrera. Health Nutrition and Population Global Practice, World Bank, Santiago, Chile. cherrerariquelme@worldbank.org

Jeremy J. Hess. Center for Health and the Global Environment, University of Washington, Seattle, WA, USA. jjhess@uw.edu

Zuhelen Padilla. Pan American Health Organization, Washington, DC, USA. padillazuh@paho.org

Karen Polson. Pan American Health Organization, Washington, DC, USA. polsonkar@paho.org

Zoila Vela-Clavo. Centro Latino Americano de Excelencia en Cambio Climático y Salud, Universidad Peruana Cayetano Heredia, Lima, Peru. zoila.vela@upch.pe

Daniel F Buss. Pan American Health Organization, Washington, DC, USA. bussd@paho.org

Stella M. Hartinger. Centro Latino Americano de Excelencia en Cambio Climático y Salud, Universidad Peruana Cayetano Heredia, Lima, Peru. STELLA.HARTINGER.P@upch.pe

Section A. Components and areas to evaluate in this study following the PAHO agenda.

Governance and Intersectoral Action Structures

Multi-institutional structures on climate change

- Establishment of a multi-institutional structure to work on climate change at national level (e.g., national inter-ministerial committee, national coordination mechanism)
    - Representative of Ministry with responsibility for Human Health (MoH) participating in this structure

Focal points and multi-stakeholder mechanisms (e.g. task force or committee)

- Ministry responsible for health and/or climate change
- Designation of a focal point responsible for health and climate change at MoH
- Establishment of an operational multi-stakeholder structure to work on climate change at MoH
    - Health programmes that participate in the multi-stakeholder mechanism (selection from: health systems; noncommunicable diseases; food and nutrition; vector-borne diseases; communicable diseases; public health emergencies; zoonoses)
    - Cross-cutting areas that participate in the multi-stakeholder mechanism (selection from: equity; gender; One Health; epidemiology and surveillance; air quality/pollution; chemical safety; water, sanitation, and hygiene; occupational health; road safety; environmental health)
    - Health-determining sectors/ministries that participate in the health and climate change multi-stakeholder mechanism (selection from: agriculture; biodiversity; education; energy; environment; housing; industry; national meteorological and hydrological services; social services; transportation; urban development; water authority)
    - Stakeholders and/or experts that participate in the health and climate change multi-stakeholder mechanism (selection from: academia; civil society; community groups; indigenous groups; nongovernmental organisations; private sector; local government)

Intersectoral memorandum of understanding

- Existence of joint memorandum of understanding or another agreement in place between MoH and this sector/ministry (selection from: agriculture; education; energy; environment; urban development/housing; national meteorological and hydrological services; social services; transportation; water, sanitation, and hygiene).

Planning and Regulatory Frameworks

National Health report

- Inclusion of climate change and health in the most recent national health report or equivalent

Vulnerability and adaptation assessments

- Performance of vulnerability and adaptation assessments (including year of completion, level of coverage, population groups considered)
- Results of the most recent assessment in the development of new health policies/programmes or the revision of existing health policies/programmes

- Influence of the assessment in allocation of human and financial resources
- Health and climate change topics prioritised in the national health and climate change plan/strategy

Health and climate change plan/strategy

- National health and climate change plan/strategy
    - Description of: year of completion; time period covered; leading institution; part of NAP, national plan for poverty reduction or national development plan, national portfolio of actions on environment and health, a situation analysis and needs assessment
    - Strategy informed by vulnerability and adaptation assessment
    - Health and climate change topics prioritised in the plan/strategy
    - Health and climate change actions prioritised in the plan/strategy
    - Estimation of the budget and human resources required to implement the plan/strategy

Health Surveillance and Integrated Information Systems

Health surveillance

- Health surveillance system includes meteorological information: For the climate sensitive health risks/outcomes: air-borne and respiratory illnesses; heat-related illness; injury and mortality from extreme weather events; malnutrition and food-borne diseases; mental and psychosocial health; noncommunicable diseases; vector-borne diseases; water-borne diseases and other water-related health outcomes; zoonoses; impacts on health facilities.
- Climate-informed health early warning system in place: For the climate sensitive health risks/outcomes: air-borne and respiratory illnesses; heat-related illness; injury and mortality from extreme weather events; malnutrition and food-borne diseases; mental and psychosocial health; noncommunicable diseases; vector-borne diseases; water-borne diseases and other water-related health outcomes; zoonoses; impacts on health facilities
- Health sector response plan in place: For the climate sensitive health risks/outcomes: air-borne and respiratory illnesses; heat-related illness; injury and mortality from extreme weather events; malnutrition and food-borne diseases; mental and psychosocial health; noncommunicable diseases; vector-borne diseases; water-borne diseases and other water-related health outcomes; zoonoses; impacts on health facilities
- Mechanism to communicate early warnings to the general population in place: For the climate sensitive health risks/outcomes: air-borne and respiratory illnesses; heat-related illness; injury and mortality from extreme weather events; malnutrition and food-borne diseases; mental and psychosocial health; noncommunicable diseases; vector-borne diseases; water-borne diseases and other water-related health outcomes; zoonoses; impacts on health facilities

Early warning systems in place

- For climate-related events: heatwaves; cold waves; storms; flooding; landslides/mudslides; sea level rise; fire; droughts; air quality; sand/dust storms.

Use of information

- Use of information from any regional or national integrated data platforms to inform health and climate change operational decisions and programmes

Climate and Health Financing

Funding award

- MoH receives international funds to support climate change and health work.
    - Description of: Total amount and currency; time period covered; purpose/focus of the funding

Capacity

- Personnel at MoH responsible for management of climate change and health related projects and/or activities, include number of people.
- Personnel at MoH responsible for management of climate change and health related funds, include number of people.

National budget

- Proportion of national budget allocated for climate change and health personnel, programmes, and actions

Challenges

Greatest challenges the MoH has faced in accessing international funds for climate and health work

Primary Care and Health Infrastructure

Greenhouse gas emissions

- National target and/or recommendations for greenhouse gas emission reductions in the health sector

Climate resilient health facilities

- Public health care facilities assessed for climate resilience according to PAHO's Hospital Safety Index and Green Checklist
    - Number of public health care facilities assessed

Environmentally sustainable health facilities

- Public health care facilities assessed for environmental sustainability
    - Number of public health care facilities assessed

Clean, Healthy and Sustainable Environments

- Use of tools to assess health co-benefits of climate change action
    - Selection from: CarbonH; AirQ+; GreenUR; HEAT; other
    - Influence of findings from the application of tools on projects or policy decision-making

Emergency Preparedness and Response

- Plans and procedures for weather and climate-related disaster preparedness, response, and recovery
- Implementation of national public health communication campaigns on climate change and health to increase the general public's understanding on this topic by MoH

Research and Capacity Building

- MoH staff trained on the linkages between climate change and health over the past two years
  - Category of personnel (selection from: environmental health personnel; epidemiology/surveillance personnel; health emergency personnel; health personnel; planning personnel)
- Inclusion of climate change and its connection to human health on primary, secondary, tertiary education

Section B. Guiding questions for semi-structured interviews

Argentina (Script in original language: Spanish)

1) Entendemos que Argentina obtuvo un proyecto *readiness*, ¿Podrías contarnos cómo fue el proceso de preparación y presentación de la propuesta al Green Climate Fund, y cuáles fueron los elementos centrales de la estrategia?
   a. Pregunta de seguimiento: ¿Por qué se priorizaron esos elementos por sobre otros?
2) ¿Qué sectores (además de salud) participaron en la planificación e implementación del proyecto financiado, y cómo ha sido esa experiencia colaborativa?
   a. Pregunta de seguimiento: ¿Qué desafíos aparecieron durante la planificación e implementación del proyecto?
   b. Pregunta de seguimiento: ¿Qué aprendizajes tuvieron a partir del proceso que pudieran ser relevantes para otros países de la región?
3) En términos de sostenibilidad y escalabilidad, ¿qué acciones o políticas se proyectan para mantener y ampliar los beneficios logrados a través del financiamiento climático?

Argentina (Script in English)

1) We understand that Argentina was awarded a readiness project. Could you tell us about the process of preparing and submitting the proposal to the Green Climate Fund, and what were the core elements of the strategy?
   a. Following up question: Why were these elements prioritised over others?
2) Which sectors (besides health) were involved in the planning and implementation of the funded project, and what has that collaborative experience been like?
   a. Following up question: What challenges arose during the project's planning and implementation?
   b. Following up question: What lessons were learned from the process that could be relevant to other countries in the region?
3) In terms of sustainability and scalability, what actions or policies are planned to maintain and expand the benefits achieved through climate finance?

Chile (Script in original language: Spanish)

1) Hemos observado que Chile cuenta con un sistema robusto de manejo de determinantes de la salud medioambientales, incluyendo monitoreo y medidas de acción en contaminación del aire, calidad del agua, inicuidad alimentaria, manejo de basura y residuos, entre otros, lo que es clave para el fortalecimiento de los sistemas de salud resilientes al clima. ¿Podrías comentar cómo ha sido el proceso de generación de estas medidas e integración de los determinantes ambientales en las políticas públicas en salud en Chile?
    a. Pregunta de seguimiento: ¿Hasta qué medida sectores fuera del sector salud (por ejemplo medioambiente, energía, transporte, servicios meteorológicos) contribuyen al monitoreo y manejo de estos determinantes?
2) En tu experiencia, ¿cuáles han sido los principales desafíos en el monitoreo y manejo de estos determinantes considerando la necesidad de un trabajo multisectorial?
3) ¿Hasta qué grado el monitoreo y manejo de determinantes medioambientales se integran e informan los planes de Adaptación en Salud, Plan de Mitigación y NDCs?
    a. Pregunta de seguimiento: ¿Cómo la integración ha contribuido a mejorar la resiliencia del sistema de salud?

Chile (Script in English)

1) We have observed that Chile has a robust system for managing environmental determinants of health, including monitoring and action measures for air pollution, water quality, food inequity, and waste management, among others. Could you comment on the process of integrating environmental determinants into public health policies in Chile?
    a. Following up question: To what extent do sectors outside the health sector (e.g., environment, energy, transportation, meteorological services) contribute to the monitoring and management of these determinants?
2) In your experience, what have been the main challenges in monitoring and managing these determinants, considering the need for multisectoral work?
3) To what extent are the monitoring and management of environmental determinants integrated into and informed by Health Adaptation Plans, Mitigation Plans, and NDCs?
    a. Following up question: How has this integration contributed to improving the resilience of the health system?

Jamaica (**Script in original language: English)**

1) We have identified that Jamaica has V&A assessments at the subnational level and is part of a PAHO/GCF readiness project focused on establishing operational national committees and generating baseline data through vulnerability and adaptation assessments and country profiles. Can you explain how the V&A assessments are carried out in the country and how they have informed public policy on health adaptation to climate change?
    a. Following up question: To what extend has the readiness project supported the generation of V&A assessments at the subnational level?
2) Given that V&A assessments require working with other sectors and actors, how have your experience been working with them?
    b. Following up question: Have you ever encountered challenges during this process? If so, which?
    c. Following up question: What lessons have you learnt from these processes?

Peru (Script in original language: Spanish)

1. Perú ha estado desarrollando programas de capacitación o entrenamiento para personas que trabajan en el sector salud, incluyendo en el MINSA. Nos podrías contar un poco sobre ese proyecto, cómo ha sido la experiencia, desarrollo e implementación.
    a. Pregunta de seguimiento: ¿Cuáles son las motivaciones del MINSA para llevar a cabo estas capacitaciones?
2. En relación a la implementación del proyecto, ¿a qué niveles se ha implementado (nacional, regional, municipal) y qué otros actores han estado involucrados?
    a. Pregunta de seguimiento:¿Cómo se han incorporado aspectos culturales en el proyecto o programa?
3. A modo de reflexión, ¿cuáles han sido los tres desafíos más grandes en el diseño e implementación del programa de capacitación?
    a. Pregunta de seguimiento: ¿Se han podido sobrellevar? ¿Cómo?
    b. Pregunta de seguimiento: ¿Cómo se aseguró financiamiento para esto? ¿Cómo se puede sobrellevar para el futuro? Si no aparece, preguntar.
4. ¿Cuáles han sido los tres aprendizajes en el diseño e implementación del programa de capacitación?

Peru (Script in English)

1) Peru has been developing training programs for people working in the health sector, including the Ministry of Health (MINSA). Could you tell us a little about this project, including development and implementation?
    a. Following up question: What are the Ministry of Health's motivations for carrying out these trainings?
2) Regarding the project's implementation, at what levels has it been implemented (national, regional, municipal) and what other actors have been involved?
    a. Following up question: How have cultural aspects been incorporated into the project or program?
3) As a reflection, what have been the three biggest challenges in designing and implementing the training program?
    a. Following up question: Have they been overcome? How?
    b. Following up question: How was funding secured for this? How can this be addressed in the future? If not, ask.
4) What have been the three lessons learned from the design and implementation of the training program?

Section C. Participating countries in 2021 and 2023 PAHO surveys

Table A1. Participating countries in 2021 and 2023 PAHO survey.

Region	2021*	2023**
Caribbean	Bahamas	Bahamas
	Barbados	Barbados
	Belize	Belize
	Dominica	Dominica
	Grenada	Grenada
	Guyana	Guyana
	Haiti	
	Jamaica	Jamaica
	Saint Kitts and Nevis	Saint Kitts and Nevis
	Saint Lucia	Saint Lucia
		Saint Vincent and the Grenadines
	Suriname	Suriname
	Trinidad and Tobago	Trinidad and Tobago
Central America	Costa Rica	Costa Rica
	Cuba	Cuba
	Dominican Republic	Dominican Republic
	El Salvador	El Salvador
	Guatemala	Guatemala
	Nicaragua	Nicaragua
		Panama
South America	Argentina	Argentina
	Bolivia	
	Brazil	Brazil
		Chile
	Colombia	Colombia
		Ecuador
		Paraguay
	Peru	Peru
	Uruguay	Uruguay

* Countries not participating in 2021 survey: Antigua and Barbuda; Saint Vincent and the Grenadines; Honduras; Mexico; Panama; Chile; Ecuador; Paraguay; Venezuela.

** Countries not participating in 2023 survey: Antigua and Barbuda; Haiti; Honduras; Mexico; Bolivia; Venezuela.

Section D. Tables with information respective to each question of PAHO surveys

Governance and Intersectoral Action Structures

Table A2. Has your country established a multi-institutional structure to work on climate change (e.g. national inter-ministerial committee, national coordination mechanism)?

Region	Yes n (% per sub-region)		No n (% per sub-region)		Unknown n (% per sub-region)		No response n (% per sub-region)	
	2021	2023	2021	2023	2021	2023	2021	2023
Car (n= 14)	10 (71.4)	11 (78.6)	2 (14.3)	0 (0)	0 (0)	0 (0)	2 (14.3)	3 (21.4)
CA (n=9)	6 (66.7)	7 (77.8)	0 (0)	0 (0)	0 (0)	0 (0)	3 (33.3)	2 (22.2)
SA (n=10)	5 (50)	8 (80)	0 (0)	0 (0)	1 (10)	0 (0)	4 (40)	2 (20)

Table A3. If yes, does a Ministry of Health representative participate in this multi-institutional structure on climate change?

Region	Yes n (% per sub-region)		Unknown n (% per sub-region)		No response n (% per sub-region)	
	2021	2023	2021	2023	2021	2023
Car (n= 14)	10 (71.4)	11 (78.6)	-	0 (0)	4 (28.6)	3 (21.4)
CA (n=9)	6 (66.7)	6 (66.7)	-	1 (11.1)	3 (33.3)	2 (22.2)
SA (n=10)	4 (40)	8 (80)	-	0 (0)	6 (60)	2 (20)

Table A4. Does the Ministry of Health have a designated focal point responsible for health and climate change?

Region	Yes n (% per sub-region)		No n (% per sub-region)		Unknown n (% per sub-region)		No response n (% per sub-region)	
	2021	2023	2021	2023	2021	2023	2021	2023
Car (n= 14)	11 (78.6)	13 (92.9)	1 (7.1)	0 (0)	0 (0)	0 (0)	2 (14.3)	1 (7.14)
CA (n=9)	6 (66.6)	7 (77.8)	0 (0)	0 (0)	0 (0)	0 (0)	3 (33.3)	2 (22.2)
SA (n=10)	4 (40)	8 (80)	0 (0)	0 (0)	1 (10)	0 (0)	5 (50)	2 (20)

Table A5. If the Ministry of Health established a multi-stakeholder mechanism* on health and climate change that is currently operational (e.g. task force or committee), select all health programmes that participate in the health and climate change multi-stakeholder mechanism (e.g. taskforce or committee)

Region	Yes n (% per sub-region)		No response n (% per sub-region)	
	2021	2023	2021	2023
**Health systems strengthening**				
Car (n= 14)	2 (14.3)	2 (14.3)	12 (85.7)	12 (85.7)
CA (n=9)	3 (33.3)	2 (22.2)	6 (66.7)	7 (77.8)
SA (n=10)	3 (30)	4 (40)	7 (70)	6 (60)
**Noncommunicable diseases**				
Car (n= 14)	4 (28.6)	2 (14.3)	10 (71.4)	12 (85.7)
CA (n=9)	4 (44.4)	2 (22.2)	5 (55.6)	7 (77.8)
SA (n=10)	4 (40)	4 (40)	6 (60)	6 (60)
**Food and nutrition**				
Car (n= 14)	1 (7.1)	3 (21.4)	13 (92.9)	11 (78.6)
CA (n=9)	2 (22.2)	4 (44.4)	7 (77.8)	5 (55.6)
SA (n=10)	1 (10)	3 (30)	9 (90)	7 (70)
**Vector-borne diseases**				
Car (n= 14)	4 (28.6)	4 (28.6)	10 (71.4)	10 (71.4)
CA (n=9)	4 (44.4)	4 (44.4)	5 (55.6)	5 (55.6)
SA (n=10)	4 (40)	5 (50)	6 (60)	5 (50)
**Communicable diseases**				
Car (n= 14)	-	3 (21.4)	-	11 (78.6)
CA (n=9)	-	3 (33.3)	-	6 (66.7)
SA (n=10)	-	4 (40)	-	6 (60)
**Public Health Emergencies**				
Car (n= 14)	-	4 (28.6)	-	10 (71.4)
CA (n=9)	-	4 (44.4)	-	5 (55.6)
SA (n=10)	-	5 (50)	-	5 (50)
**Zoonoses**				
Car (n= 14)	2 (14.3)	4 (28.6)	12 (85.7)	10 (71.4)
CA (n=9)	3 (33.3)	4 (44.4)	6 (66.7)	5 (55.6)
SA (n=10)	2 (20)	4 (40)	8 (80)	6 (60)

* Note: The multi-stakeholder mechanism could be either internal (health ministry only) or external (between the health ministry and other health-determining sectors, organizations and experts).

Table A6. If the Ministry of Health established a multi-stakeholder mechanism* on health and climate change that is currently operational (e.g. task force or committee), select all cross-cutting areas that participate in the health and climate change multi-stakeholder mechanism (e.g. taskforce or committee).

Region	Yes n (% per sub-region)		No response n (% per sub-region)	
	2021	2023	2021	2023
**Equity**				
Car (n= 14)	1 (7.14)	2 (14.3)	13 (92.9)	12 (85.7)
CA (n=9)	0 (0)	2 (22.2)	9 (100)	7 (77.8)
SA (n=10)	3 (30)	3 (30)	7 (70)	7 (70)
**Gender**				
Car (n= 14)	3 (21.4)	2 (14.3)	11 (78.6)	12 (85.7)
CA (n=9)	3 (33.3)	2 (22.2)	6 (66.7)	7 (77.8)
SA (n=10)	2 (20)	4 (40)	8 (80)	6 (60)
**One health**				
Car (n= 14)	0 (0)	3 (21.4)	14 (100)	11 (78.6)
CA (n=9)	1 (11.1)	2 (22.2)	8 (88.9)	7 (77.8)
SA (n=10)	1 (10)	1 (10)	9 (90)	9 (90)
**Epidemiology and surveillance**				
Car (n= 14)	-	4 (28.6)	-	10 (71.4)
CA (n=9)	-	4 (44.4)	-	5 (55.6)
SA (n=10)	-	5 (50)	-	5 (50)
**Air quality/pollution**				
Car (n= 14)	2 (14.3)	2 (14.3)	12 (85.7)	12 (85.7)
CA (n=9)	1 (11.1)	4 (44.4)	8 (88.9)	5 (55.6)
SA (n=10)	3 (30)	4 (40)	7 (70)	6 (60)
**Chemical safety**				
Car (n= 14)	1 (7.1)	3 (21.4)	13 (92.9)	11 (78.6)
CA (n=9)	1 (11.1)	3 (33.3)	8 (88.9)	6 (66.7)
SA (n=10)	1 (10)	2 (20)	9 (90)	8 (80)
**Water, sanitation and hygiene (WASH)**				
Car (n= 14)	2 (14.3)	4 (28.6)	12 (85.7)	10 (71.4)
CA (n=9)	3 (33.3)	4 (44.4)	6 (66.7)	5 (55.6)
SA (n=10)	2 (20)	4 (40)	8 (80)	6 (60)
**Occupational health**				
Car (n= 14)	1 (7.1)	3 (21.4)	13 (92.9)	11 (78.6)
CA (n=9)	1 (11.1)	4 (44.4)	8 (88.9)	5 (55.6)
SA (n=10)	0 (0)	2 (20)	10 (100)	8 (80)
**Road Safety**				
Car (n= 14)	4 (28.6)	1 (7.14)	10 (71.4)	13 (92.9)
CA (n=9)	4 (44.4)	1 (11.1)	5 (55.6)	8 (88.9)
SA (n=10)	3 (30)	1 (10)	7 (70)	9 (90)
**Environmental Health**				
Car (n= 14)	4 (28.6)	4 (28.6)	10 (71.4)	10 (71.4)
CA (n=9)	3 (33.3)	4 (44.4)	6 (66.7)	5 (55.6)
SA (n=10)	1 (10)	5 (50)	9 (90)	5 (50)

Table A7. If the Ministry of Health established a multi-stakeholder mechanism* on health and climate change that is currently operational (e.g. task force or committee), select all health-determining sectors/ministries that participate in the health and climate change multi-stakeholder mechanism (e.g. taskforce or committee).

Region	Yes n (% per sub-region)		No response n (% per sub-region)	
	2021	2023	2021	2023
**Agriculture**				
Car (n= 14)	0 (0)	4 (28.6)	14 (100)	10 (71.4)
CA (n=9)	1 (11.1)	4 (44.4)	8 (88.9)	5 (55.6)
SA (n=10)	1 (10)	4 (40)	9 (90)	6 (60)
**Biodiversity**				
Car (n= 14)	1 (7.1)	1 (7.1)	13 (92.9)	13 (92.9)
CA (n=9)	2 (22.2)	2 (22.2)	7 (77.8)	7 (77.8)
SA (n=10)	1 (10)	2 (20)	9 (90)	8 (80)
**Education**				
Car (n= 14)	2 (14.3)	2 (14.3)	12 (85.7)	12 (85.7)
CA (n=9)	0 (0)	2 (22.2)	9 (100)	7 (77.8)
SA (n=10)	1 (10)	2 (20)	9 (90)	8 (80)
**Energy**				
Car (n= 14)	3 (21.4)	1 (7.1)	11 (78.6)	13 (92.9)
CA (n=9)	3 (33.3)	2 (22.2)	6 (66.7)	7 (77.8)
SA (n=10)	3 (30)	3 (30)	7 (70)	7 (70)
**Environment**				
Car (n= 14)	0 (0)	3 (21.4)	14 (100)	11 (78.6)
CA (n=9)	1 (11.1)	3 (33.3)	8 (88.9)	6 (66.7)
SA (n=10)	0 (0)	5 (50)	10 (100)	5 (50)
**Housing**				
Car (n= 14)	0 (0)	2 (14.3)	14 (100)	12 (85.7)
CA (n=9)	1 (11.1)	2 (22.2)	8 (88.9)	7 (77.8)
SA (n=10)	1 (10)	1 (10)	9 (90)	9 (90)
**Industry**				
Car (n= 14)	3 (21.4)	1 (7.1)	11 (78.6)	13 (92.9)
CA (n=9)	2 (22.2)	2 (22.2)	7 (77.8)	7 (77.8)
SA (n=10)	3 (30)	2 (20)	7 (70)	8 (80)
**National meteorological and hydrological services**				
Car (n= 14)	1 (7.1)	3 (21.4)	13 (92.9)	11 (78.6)
CA (n=9)	1 (11.1)	3 (33.3)	8 (88.9)	6 (66.7)
SA (n=10)	0 (0)	4 (40)	10 (100)	6 (60)
**Social services**				
Car (n= 14)	2 (14.3)	2 (14.3)	12 (85.7)	12 (85.7)
CA (n=9)	1 (11.1)	1 (11.1)	8 (88.9)	8 (88.9)
SA (n=10)	1 (10)	1 (10)	9 (90)	9 (90)
**Transportation**				
Car (n= 14)	0 (0)	1 (7.1)	14 (100)	13 (92.9)
CA (n=9)	1 (11.1)	1 (11.1)	8 (88.9)	8 (88.9)
SA (n=10)	1 (10)	2 (20)	9 (90)	8 (80)
**Urban development**				
Car (n= 14)	3 (21.4)	1 (7.1)	11 (78.6)	13 (92.9)
CA (n=9)	3 (33.3)	2 (22.2)	6 (66.7)	7 (77.8)
SA (n=10)	4 (40)	1 (10)	6 (60)	9 (90)
**Water authority**				
Car (n= 14)	-	2 (14.2)	-	12 (85.7)
CA (n=9)	-	1 (11.1)	-	8 (88.9)
SA (n=10)	-	3 (30)	-	7 (70)

Table A8. If the Ministry of Health established a multi-stakeholder mechanism* on health and climate change that is currently operational (e.g. task force or committee), select all stakeholders and/or experts that participate in the health and climate change multi-stakeholder mechanism (e.g. taskforce or committee).

Region	Yes n (% per sub-region)		No response n (% per sub-region)	
	2021	2023	2021	2023
**Academia**				
Car (n= 14)	2 (14.3)	3 (21.4)	12 (85.7)	11 (78.6)
CA (n=9)	1 (11.1)	3 (33.3)	8 (88.9)	6 (66.7)
SA (n=10)	5 (50)	5 (50)	5 (50)	5 (50)
**Civil society**				
Car (n= 14)	2 (14.3)	2 (14.3)	12 (85.7)	12 (85.7)
CA (n=9)	0 (0)	2 (22.2)	9 (100)	7 (77.8)
SA (n=10)	1 (10)	4 (40)	9 (90)	6 (60)
**Community groups**				
Car (n= 14)	1 (7.1)	2 (14.3)	13 (92.9)	12 (85.7)
CA (n=9)	1 (11.1)	2 (22.2)	8 (88.9)	7 (77.8)
SA (n=10)	1 (10)	2 (20)	9 (90)	8 (80)
**Indigenous groups**				
Car (n= 14)	0 (0)	1 (7.1)	14 (100)	13 (92.9)
CA (n=9)	0 (0)	1 (11.1)	9 (100)	8 (88.9)
SA (n=10)	1 (10)	1 (10)	9 (90)	9 (90)
**Nongovernmental organizations (NGOs)**				
Car (n= 14)	3 (21.4)	2 (14.3)	11 (78.6)	12 (85.7)
CA (n=9)	0 (0)	3 (33.3)	9 (100)	6 (66.7)
SA (n=10)	1 (10)	4 (40)	9 (90)	6 (60)
**Private sector**				
Car (n= 14)	1 (7.1)	2 (14.3)	13 (92.9)	12 (85.7)
CA (n=9)	0 (0)	3 (33.3)	9 (100)	6 (66.7)
SA (n=10)	0 (0)	3 (30)	10 (100)	7 (70)
**Local government (e.g. state, district, and municipal government)**				
Car (n= 14)	1 (7.1)	1 (7.1)	13 (92.9)	13 (92.9)
CA (n=9)	2 (22.2)	3 (33.3)	7 (77.8)	6 (66.7)
SA (n=10)	4 (40)	5 (50)	6 (60)	5 (50)

Table A9. For the following health determining sectors, please indicate if there is a joint memorandum of understanding or other agreement in place between the Ministry of Health and this sector/ministry which defines specific roles and responsibilities in relation to health and climate change policy or programs.

Region	Yes n (% per sub-region)		No n (% per sub-region)		Unknown n (% per sub-region)		No response n (% per sub-region)	
	2021	2023	2021	2023	2021	2023	2021	2023
**Agriculture**								
Car (n= 14)	0 (0)	0 (0)	10 (71.4)	11 (78.6)	1 (7.1)	1 (7.1)	3 (21.4)	2 (14.3)
CA (n=9)	2 (22.2)	1 (11.1)	3 (33.3)	3 (33.3)	1 (11.1)	2 (22.2)	3 (33.3)	3 (33.3)
SA (n=10)	0 (0)	2 (20)	6 (60)	5 (50)	0 (0)	1 (10)	4 (40)	2 (20)
**Education**								
Car (n= 14)	0 (0)	0 (0)	10 (71.4)	11 (78.6)	1 (7.1)	1 (7.1)	3 (21.4)	2 (14.3)
CA (n=9)	2 (22.2)	1 (11.1)	3 (33.3)	3 (33.3)	1 (11.1)	2 (22.2)	3 (33.3)	3 (33.3)
SA (n=10)	0 (0)	2 (20)	5 (50)	5 (50)	1 (10)	1 (10)	4 (40)	2 (20)
**Energy**								
Car (n= 14)	0 (0)	0 (0)	9 (64.3)	11 (78.6)	2 (14.3)	1 (7.1)	3 (21.4)	2 (14.3)
CA (n=9)	2 (22.2)	1 (11.1)	3 (33.3)	3 (33.3)	1 (11.1)	2 (22.2)	3 (33.3)	3 (33.3)
SA (n=10)	0 (0)	3 (30)	6 (60)	4 (40)	0 (0)	1 (10)	4 (40)	2 (20)
**Environment**								
Car (n= 14)	2 (14.3)	1 (7.1)	9 (64.3)	10 (71.4)	1 (7.1)	1 (7.1)	2 (14.3)	2 (14.3)
CA (n=9)	2 (22.2)	3 (33.3)	3 (33.3)	2 (22.2)	1 (11.1)	2 (22.2)	3 (33.3)	2 (22.2)
SA (n=10)	1 (10)	2 (20)	4 (40)	4 (40)	1 (10)	1 (10)	4 (40)	3 (30)
**Urban development/Housing**								
Car (n= 14)	1 (7.1)	0 (0)	9 (64.3)	9 (64.3)	1 (7.1)	1 (7.1)	3 (21.4)	4 (28.6)
CA (n=9)	1 (11.1)	1 (11.1)	3 (33.3)	3 (21.4)	2 (22.2)	2 (22.2)	3 (33.3)	3 (33.3)
SA (n=10)	0 (0)	2 (20)	5 (50)	5 (50)	1 (10)	1 (10)	4 (40)	2 (20)
**National meteorological and hydrological services**								
Car (n= 14)	0 (0)	1 (7.1)	10 (71.4)	8 (57.1)	1 (7.1)	3 (21.4)	3 (21.4)	2 (14.3)
CA (n=9)	2 (22.2)	2 (22.2)	3 (33.3)	2 (22.2)	1 (11.1)	2 (22.2)	3 (33.3)	3 (33.3)
SA (n=10)	1 (10)	3 (30)	5 (50)	4 (40)	0 (0)	1 (10)	4 (40)	2 (20)
**Social services**								
Car (n= 14)	0 (0)	0 (0)	10 (71.4)	11 (78.6)	1 (7.1)	1 (7.1)	3 (21.4)	2 (14.3)
CA (n=9)	0 (0)	1 (11.1)	4 (44.4)	3 (33.3)	1 (11.1)	2 (22.2)	4 (44.4)	3 (33.3)
SA (n=10)	0 (0)	2 (20)	5 (50)	5 (50)	1 (10)	1 (10)	4 (40)	2 (20)
**Transportation**								
Car (n= 14)	0 (0)	0 (0)	10 (71.4)	9 (64.3)	1 (7.1)	1 (7.1)	3 (21.4)	4 (28.6)
CA (n=9)	1 (11.1)	0 (0)	3 (33.3)	3 (33.3)	1 (11.1)	2 (22.2)	4 (44.4)	4 (44.4)
SA (n=10)	0 (0)	2 (20)	5 (50)	5 (50)	1 (10)	1 (10)	4 (40)	2 (20)
**Water, sanitation & hygiene (WASH)**								
Car (n= 14)	2 (14.3)	0 (0)	7 (50)	8 (57.1)	1 (7.1)	1 (7.1)	4 (28.6)	5 (35.7)
CA (n=9)	2 (22.2)	3 (33.3)	2 (22.2)	2 (22.2)	1 (11.1)	2 (22.2)	4 (44.4)	2 (22.2)
SA (n=10)	0 (0)	2 (20)	6 (60)	5 (50)	0 (0)	1 (10)	4 (40)	2 (20)

Planning and Regulatory Frameworks

Table A10. Is Climate Change and Health included in the most recent National Health Report or equivalent documents?

Region	Yes n (% per sub-region)		No n (% per sub-region)		Unknown n (% per sub-region)		No response n (% per sub-region)	
	2021	2023	2021	2023	2021	2023	2021	2023
Car (n= 14)	-	1 (7.1)	-	8 (57.1)	-	3 (21.4)	-	2 (14.3)
CA (n=9)	-	4 (44.4)	-	1 (11.1)	-	2 (22.2)	-	2 (22.2)
SA (n=10)	-	4 (40)	-	3 (30)	-	1 (10)	-	2 (20)

Table A11. Is there a national health and climate change plan/strategy in place?*

Region	Yes n (% sub-region)		Under Development n (% sub-region)		No n (% sub-region)		Unknown n (% sub-region)		No response n (% sub-region)	
	2021	2023	2021	2023	2021	2023	2021	2023	2021	2023
Car (n= 14)	0 (0)	7 (50)	5 (35.7)	2 (14.3)	7 (50)	4 (28.6))	-	0 (0)	2 (14.3)	1 (7.1)
CA (n=9)	1 (11.1)	2 (22.2)	3 (33.3)	3 (33.3)	2 (22.2)	2 (22.2)	-	0 (0)	3 (33.3)	2 (22.2)
SA (n=10)	1 (10)	4 (40)	3 (30)	3 (30)	2 (20)	0 (0)	-	1 (10)	4 (40)	2 (20)

* Definition: In this questionnaire, a national health and climate change plan/strategy is a government plan or strategy which considers the health risks of climate change, and health adaptation and/or health resilience to climate change. It could be part of a broader national climate change plan/strategy that includes health.

Table A12. Has your country conducted a climate change and health vulnerability and adaptation assessment(s)?*

Region	Yes n (% sub-region)		Under Development n (% sub-region)		No n (% sub-region)		Unknown n (% sub-region)		No response n (% sub-region)	
	2021	2023	2021	2023	2021	2023	2021	2023	2021	2023
Car (n= 14)	3 (21.4)	5 (35.5)	2 (14.3)	3 (21.4)	7 (50)	3 (21.4)	0 (0)	1 (7.1)	2 (14.3)	2 (14.3)
CA (n=9)	2 (22.2)	2 (22.2)	2 (22.2)	4 (44.4)	1 (11.1)	1 (11.1)	1 (11.1)	0 (0)	3 (33.3)	2 (22.2)
SA (n=10)	1 (10)	3 (30)	2 (20)	3 (30)	3 (30)	1 810)	0 (0)	1 (10)	4 (40)	2 (20)

* Definition: A vulnerability and adaptation assessment is a process and a tool that allows countries to evaluate which populations are most vulnerable to different kinds of health effects from climate change, to identify weaknesses in the systems that should protect them, and to specify interventions to respond. Assessments can also improve evidence and understanding of the linkages between climate and health within the assessment area, serve as a baseline analysis against which changes in disease risk and protective measures can be monitored, provide the opportunity for building capacity, and strengthen the case for investment in health protection. NOTE: It is possible that multiple assessments have been conducted. For example, at national or subnational levels

Table A13. Please provide the year of completion/publication of the most recent assessment (only 2023)

Year<br>n (% sub-region)	Car (n= 14)	CA (n=9)	SA (n=10)
**2015**	1 (7.1)	0 (0)	0 (0)
**2016**	0 (0)	0 (0)	0 (0)
**2017**	0 (0)	0 (0)	0 (0)
**2018**	0 (0)	0 (0)	0 (0)
**2019**	1 (7.1)	0 (0)	1 (10)
**2020**	0 (0)	0 (0)	1 (10)
**2021**	1 (7.1)	1 (11.1)	0 (0)
**2022**	2 (14.3)	0 (0)	0 (0)
**2023**	1 (7.1)	1 (11.1)	0 (0)
**2024**	0 (0)	0 (0)	1 (10)
**No response**	8 (57.1)	7 (77.8)	7 (70)

Table A14. Please provide the level of coverage of the most recent assessment (only 2023 survey)

Region	National<br>n (% sub-region)	Sub-national<br>n (% sub-region)	District<br>n (% sub-region)	No response<br>n (% sub-region)
Car (n= 14)	4 (28.6)	2 (14.3)	0 (0)	8 (57.1)
CA (n=9)	2 (22.2)	0 (0)	0 (0)	7 (77.8)
SA (n=10)	2 (20)	0 (0)	1 (10)	7 (70)

Table A15. Please indicate specific population groups that were considered in the most recent assessment. Select all that apply (only 2023).

Region	Yes<br>n (% sub-region)	No response<br>n (% sub-region)
**Children**		
Car (n= 14)	4 (28.6)	10 (71.4)
CA (n=9)	2 (22.2)	7 (77.8)
SA (n=10)	0 (0)	10 (100)
**Displaced or migrant population**		
Car (n= 14)	2 (14.3)	12 (85.7)
CA (n=9)	0 (0)	9 (100)
SA (n=10)	0 (0)	10 (100)
**The elderly (65+ years of age)**		
Car (n= 14)	5 (35.7)	9 (64.3)
CA (n=9)	2 (22.2)	7 (77.8)
SA (n=10)	1 (10)	9 (90)
**Indigenous groups**		
Car (n= 14)	1 (7.1)	13 (92.9)
CA (n=9)	1 (11.1)	8 (88.9)
SA (n=10)	1 (10)	9 (90)
**Populations living in poverty**		
Car (n= 14)	4 (28.6)	10 (71.4)
CA (n=9)	1 (11.1)	8 (88.9)
SA (n=10)	1 (10)	9 (90)
**Women**		
Car (n= 14)	5 (35.7)	9 (64.3)
CA (n=9)	2 (22.2)	7 (77.8)
SA (n=10)	0 (0)	10 (100)
**Workers**		
Car (n= 14)	4 (28.6)	10 (71.4)
CA (n=9)	2 (22.2)	7 (77.8)
SA (n=10)	1 (10)	9 (90)
**Rural populations**		
Car (n= 14)	3 (21.4)	11 (78.6)
CA (n=9)	2 (22.2)	7 (77.8)
SA (n=10)	0 (0)	10 (100)
**Urban/peri-urban populations**		
Car (n= 14)	1 (7.1)	13 (92.9)
CA (n=9)	2 (22.2)	7 (77.8)
SA (n=10)	1 (10)	9 (90)

Table A16. If you have conducted a climate change and health vulnerability and adaptation assessment(s), were the results of the assessment(s) used to inform the development of the national health and climate change plan/strategy?

Region	Yes<br>n (% sub-region)		No<br>n (% sub-region)		Not applicable<br>n (% sub-region)		No response<br>n (% sub-region)	
	2021	2023	2021	2023	2021	2023	2021	2023
Car (n= 14)	0 (0)	2 (14.3)	-	0 (0)	3 (21.4)	1 (7.1)	11 (78.6)	11 (78.6)
CA (n=9)	0 (0)	1 (11.1)	-	0 (0)	1 (11.1)	1 (11.1)	8 (88.9)	7 (77.8)
SA (n=10)	1 (10)	2 (20)	-	1 (10)	1 (10)	1 (10)	8 (80)	6 (60)

Table A17. Did the results of the most recent assessment result in the development of new health policies or programs OR the revision of existing health policies and/or programs? (2023)

Region	Strongly n (% sub-region)	Moderately n (% sub-region)	Minimally n (% sub-region)	No n (% sub-region)	Unknown n (% sub-region)	No response n (% sub-region)
Car (n= 14)	1 (7.1)	0 (0)	3 (21.4)	1 (7.1)	1 (7.1)	8 (57.1)
CA (n=9)	0 (0)	0 (0)	1 (11.1)	1 (1.11)	0 (0)	7 (77.8)
SA (n=10)	1 (10)	1 (10)	0 (0)	0 (0)	1 (10)	7 (70)

Table A18. Did the results of the assessment influence the allocation of human and financial resources within the Ministry of Health to address health risks of climate change? (2023)

Region	Strongly n (% sub-region)	Moderately n (% sub-region)	Minimally n (% sub-region)	No n (% sub-region)	Unknown n (% sub-region)	No response n (% sub-region)
Car (n= 14)	1 (7.1)	0 (0)	2 (14.3)	3 (21.4)	0 (0)	8 (57.1)
CA (n=9)	0 (0)	0 (0)	1 (11.1)	1 (11.1)	0 (0)	7 (77.8)
SA (n=10)	0 (0)	2 (10)	0 (0)	0 (0)	1 (10)	7 (70)

Table A19. For the following climate sensitive health risks/outcomes, please indicate if the health surveillance system includes meteorological information.

Region	Yes n (% per sub-region)		No n (% per sub-region)		Unknown n (% per sub-region)		No response n (% per sub-region)	
	2021	2023	2021	2023	2021	2023	2021	2023
**Air-borne and respiratory illnesses**								
Car (n= 14)	0 (0)	4 (28.6)	10 (71.4)	6 (28.6)	1 (7.1)	1 (7.1)	3 (21.4)	3 (21.4)
CA (n=9)	2 (22.2)	3 (33.3)	2 (22.2)	3 (33.3)	1 (11.1)	1 (11.1)	4 (44.4)	2 (22.2)
SA (n=10)	2 (20)	4 (40)	3 (30)	4 (40)	0 (0)	0 (0)	5 (50)	2 (20)
**Heat-related illnesses**								
Car (n= 14)	0 (0)	3 (21.4)	8 (57.1)	7 (50)	3 (21.4)	1 (7.1)	3 (21.4)	3 (21.4)
CA (n=9)	1 (11.1)	2 (22.2)	4 (44.4)	3 (33.3)	1 (11.1)	2 (22.2)	3 (33.3)	2 (22.2)
SA (n=10)	1 (10)	4 (40)	4 (40)	4 (40)	0 (0)	0 (0)	5 (50)	2 (20)
**Injury and mortality from extreme weather events**								
Car (n= 14)	0 (0)	2 (14.3)	8 (57.1)	8 (57.1)	3 (21.4)	1 (7.1)	3 (21.4)	3 (21.4)
CA (n=9)	1 (11.1)	3 (33.3)	2 (22.2)	2 (22.2)	2 (22.2)	2 (22.2)	4 (44.4)	2 (22.2)
SA (n=10)	2 (20)	2 (20)	3 (30)	4 (40)	0 (0)	2 (20)	5 (50)	2 (20)
**Malnutrition and food-borne diseases**								
Car (n= 14)	0 (0)	2 (14.3)	9 (64.3)	8 (57.1)	2 (14.3)	1 (7.1)	3 (21.4)	3 (21.4)
CA (n=9)	1 (11.1)	2 (22.2)	3 (33.3)	2 (22.2)	1 (11.1)	3 (33.3)	4 (44.4)	2 (22.2)
SA (n=10)	0 (0)	1 (10)	5 (50)	6 (60)	1 (10)	1 (10)	4 (40)	2 (20)
**Mental and psychosocial health**								
Car (n= 14)	0 (0)	2 (14.3)	8 (57.1)	8 (57.1)	3 (21.4)	1 (7.1)	3 (21.4)	3 (21.4)
CA (n=9)	1 (11.1)	1 (11.1)	3 (33.3)	3 (33.3)	1 (11.1)	3 (33.3)	4 (44.4)	2 (22.2)
SA (n=10)	0 (0)	0 (0)	4 (40)	7 (70)	1 (10)	1 (10)	5 (50)	2 (20)
**Noncommunicable diseases (NCDs)**								
Car (n= 14)	0 (0)	2 (14.3)	8 (57.1)	7 (50)	3 (21.4)	1 (7.1)	3 (21.4)	4 (28.6)
CA (n=9)	1 (11.1)	2 (22.2)	3 (33.3)	3 (33.3)	1 (11.1)	2 (22.2)	4 (44.4)	2 (22.2)
SA (n=10)	0 (0)	3 (30)	6 (60)	5 (50)	0 (0)	0 (0)	4 (40)	2 (20)
**Vector-borne diseases**								
Car (n= 14)	3 (24.1)	3 (21.4)	7 (50)	7 (50)	1 (7.1)	1 (7.1)	3 (24.1)	3 (24.1)
CA (n=9)	3 (33.3)	3 (33.3)	2 (22.2)	2 (22.2)	1 (11.1)	2 (22.2)	3 (33.3)	2 (22.2)
SA (n=10)	1 (10)	6 (60)	5 (50)	2 (20)	0 (0)	0 (0)	4 (40)	2 (20)
**Water-borne diseases and other water-related health outcomes**								
Car (n= 14)	1 (7.1)	3 (24.1)	9 (64.3)	7 (50)	1 (7.1)	1 (7.1)	3 (24.1)	3 (24.1)
CA (n=9)	3 (33.3)	3 (33.3)	2 (22.2)	2 (22.2)	1 (11.1)	2 (22.2)	3 (33.3)	2 (22.2)
SA (n=10)	0 (0)	3 (30)	6 (60)	5 (50)	0 (0)	0 (0)	4 (40)	2 (20)
**Zoonoses**								
Car (n= 14)	1 (7.1)	2 (14.3)	8 (57.1)	8 (57.1)	2 (14.3)	1 (7.1)	3 (21.4)	3 (21.4)
CA (n=9)	2 (22.2)	3 (33.3)	2 (22.2)	2 (22.2)	2 (22.2)	2 (22.2)	3 (33.3)	2 (22.2)
SA (n=10)	0 (0)	3 (30)	5 (50)	4 (40)	1 (10)	1 (10)	4 (40)	2 (20)
**Impacts on health care facilities**								
Car (n= 14)	0 (0)	2 (14.3)	9 (64.3)	7 (50	2 (14.3)	2 (14.3)	3 (21.4)	3 (21.4)
CA (n=9)	2 (22.2)	1 (11.1)	2 (22.2)	1 (11.1)	2 (22.2)	4 (44.4)	3 (33.3)	3 (33.3)
SA (n=10)	0 (0)	0 (0)	5 (50)	6 (60)	0 (0)	1 (10)	5 (50)	3 (30)

Table A20. For the following climate-related events, please indicate if there is an early warning system in place (only 2023).

Region	Yes<br>n (% per sub-region)	No<br>n (% per sub-region)	Unknown<br>n (% per sub-region)	No response<br>n (% per sub-region)
**Cold waves**				
Car (n= 14)	1 (7.1)	8 (57.1)	2 (14.3)	3 (21.4)
CA (n=9)	2 (22.2)	4 (44.4)	1 (11.1)	2 (22.2)
SA (n=10)	5 (50)	3 (30)	0 (0)	2 (20)
**Storms (e.g. hurricanes)**				
Car (n= 14)	9 (64.3)	1 (7.1)	1 (7.1)	3 (21.4)
CA (n=9)	4 (44.4)	2 (22.2)	1 (11.1)	2 (22.2)
SA (n=10)	4 (40)	4 (40)	0 (0)	2 (20)
**Flooding**				
Car (n= 14)	9 (64.3)	1 (7.1)	1 (7.1)	3 (21.4)
CA (n=9)	4 (44.4)	2 (22.2)	1 (11.1)	2 (22.2)
SA (n=10)	5 (50)	3 (30)	0 (0)	2 (20)
**Landslides/mudslides**				
Car (n= 14)	3 (21.4)	7 (50)	1 (7.1)	3 (21.4)
CA (n=9)	2 (22.2)	4 (44.4)	1 (11.1)	2 (22.2)
SA (n=10)	2 (20)	5 (50)	1 (10)	2 (20)
**Sea level rise**				
Car (n= 14)	4 (28.6)	5 (35.7)	2 (14.3)	3 (21.4)
CA (n=9)	3 (33.3)	3 (33.3)	1 (11.1)	2 (22.2)
SA (n=10)	2 (20)	4 (40)	2 (20)	2 (20)
**Fire (forest fires)**				
Car (n= 14)	3 (21.4)	6 (42.9)	2 (14.3)	3 (21.4)
CA (n=9)	3 (33.3)	3 (33.3)	1 (11.1)	2 (22.2)
SA (n=10)	5 (50)	3 (30)	0 (0)	2 (20)
**Drought**				
Car (n= 14)	8 (57.1)	2 (14.3)	1 (7.1)	3 (21.4)
CA (n=9)	3 (33.3)	1 (11.1)	2 (22.2)	2 (33.3)
SA (n=10)	4 (40)	4 (40)	0 (0)	2 (20)
**Air quality (e.g. particulate matter, ozone levels)**				
Car (n= 14)	3 (21.4)	5 (35.7)	3 (21.4)	3 (21.4)
CA (n=9)	2 (22.2)	4 (44.4)	1 (11.1)	2 (22.2)
SA (n=10)	3 (30)	5 (50)	0 (0)	2 (20)
**Sand/dust storms**				
Car (n= 14)	5 (35.7)	4 (28.6)	5 (35.7)	3 (21.4)
CA (n=9)	2 (22.2)	4 (44.4)	2 (22.2)	2 (22.2)
SA (n=10)	1 (10)	5 (50)	1 (10)	2 (20)

Climate and Health Financing

Table A21. Is the Ministry of Health currently receiving international funds to support climate change and health work?

Region	Yes n (% per sub-region)		No n (% per sub-region)		Unknown n (% per sub-region)		No response n (% per sub-region)	
	2021	2023	2021	2023	2021	2023	2021	2023
Car (n= 14)	6 (42.9)	7 (50)	3 (21.4)	4 (28.6)	3 (21.4)	1 (7.1)	2 (14.3)	2 (14.3)
CA (n=9)	1 (11.1)	1 (11.1)	3 (33.3)	4 (44.4)	2 (22.2)	2 (22.2)	3 (33.3)	2 (22.2)
SA (n=10)	1 (10)	2 (20)	2 (20)	5 (50)	1 (10)	1 (10)	6 (60)	2 (20)

Table A22. What have been the greatest challenges the Ministry of Health has faced in accessing international funds for climate and health work? (Multiple selection).

Region	Yes n (% per sub-region)		No response n (% per sub-region)	
	2021	2023	2021	2023
**Lack of information on the opportunities**				
Car (n= 14)	9 (64.3)	6 (42.9)	5 (35.7)	8 (57.1)
CA (n=9)	5 (55.6)	3 (33.3)	4 (44.4)	6 (66.7))
SA (n=10)	4 (40)	8 (80)	6 (60)	2 (20)
**Lack of country eligibility**				
Car (n= 14)	4 (28.6)	0 (0)	10 (71.4)	14 (100)
CA (n=9)	3 (33.3)	2 (22.2)	6 (66.7)	7 (77.8)
SA (n=10)	0 (0)	1 (10)	10 (100)	9 (90)
**Lack of connection by health actors to climate change processes**				
Car (n= 14)	8 (57.1)	7 (50)	6 (42.9)	7 (50)
CA (n=9)	5 (55.6)	3 (33.3)	5 (55.6)	6 (66.7)
SA (n=10)	4 (40)	3 (30)	6 (60)	7 (70)
**Lack of capacity to prepare country proposals**				
Car (n= 14)	9 (64.3)	11 (78.6)	5 (35.7)	3 (21.4)
CA (n=9)	5 (55.6)	3 (33.3)	4 (44.4)	6 (66.7)
SA (n=10)	3 (30)	4 (40)	7 (70)	6 (60)
**Lack of success in submitted applications**				
Car (n= 14)	1 (7.1)	2 (14.3)	13 (85.7)	12 (85.7)
CA (n=9)	4 (44.4)	2 (22.2)	5 (55.6)	7 (77.8)
SA (n=10)	0 (0)	0 (0)	10 (100)	10 (100)

Primary Care and Health infrastructure

Table A23. Have any of your country's public health care facilities been assessed according to PAHO's Hospital Safety Index and Green Checklist for climate resilience?*

Region	Yes n (% sub-region)		No n (% sub-region)		Unknown n (% sub-region)		No response n (% sub-region)	
	2021	2023	2021	2023	2021	2023	2021	2023
Car (n= 14)	7 (50)	7 (50)	4 (28.6)	3 (21.4)	0 (0)	2 (14.3)	3 (21.4)	2 (14.3)
CA (n=9)	2 (22.2)	4 (44.4)	3 (33.3)	1 (11.1)	1 (11.1)	2 (22.2)	3 (33.3)	2 (22.2)
SA (n=10)	0 (0)	2 (20)	3 (30)	5 (50)	3 (30)	1 (10)	4 (40)	2 (20)

* For the purpose of this question, assessing climate resilience of health care facilities refers to a process whereby health planners and/or health care facility managers would assess whether a health care facility is able to respond to, recover from and adapt to climate-related shocks and stresses while leveraging opportunities to enhance functions and services

Table A24. Have any of your country's public health care facilities been assessed for environmental sustainability?*

Region	Yes n (% sub-region)		No n (% sub-region)		Unknown n (% sub-region)		No response n (% sub-region)	
	2021	2023	2021	2023	2021	2023	2021	2023
Car (n= 14)	5 (35.7)	2 (14.3)	4 (28.6)	8 (57.1)	2 (14.3)	1 (7.1)	3 (21.4)	3 (21.4)
CA (n=9)	1 (11.1)	3 (33.3)	4 (44.4)	3 (33.3)	1 (11.1)	1 (11.1)	3 (33.3)	2 (22.2)
SA (n=10)	0 (0)	3 (30)	3 (30)	5 (50)	3 (30)	0 (0)	4 (40)	2 (20)

* NOTE: For the purpose of this question, assessing the environmental sustainability of health care facilities refers to assessing the environmental footprint of a health care facility with the aim to optimize the use of resources and minimizing the release of wastes while protecting and improving the health of their communities.

Table A25. Is there a national target and/or recommendations for greenhouse gas emission reductions in the health sector? (only 2023).

Region	Yes n (% sub-region)	No n (% sub-region)	Unknown n (% sub-region)	No response n (% sub-region)
Car (n= 14)	3 (21.4)	8 (57.1)	1 (7.1)	2 (14.3)
CA (n=9)	4 (44.4)	3 (33.3)	0 (0)	2 (22.2)
SA (n=10)	6 (60)	2 (20)	0 (0)	2 (20)

Clean, Healthy and Sustainable Environments

Table A26. Has your country used any of the following tools to assess health co-benefits of climate change action?

Region	CarbonH	AirQ+	GreenUR	HEAT	Other
Car (n= 14)	-	-	-	-	-
CA (n=9)	-	Cuba<br>Nicaragua	-	-	-
SA (n=10)	Argentina<br>Colombia	Argentina<br>Colombia	Argentina	Argentina	-

CarbonH: Estimates health cost savings derived from implementation of mitigation commitments from countries' NDCs. AirQ+: Quantifies the health burden and impact of air pollution. GreenUR: Quantifies the positive health impacts of implementing green spaces in urban areas. HEAT: Health Economic Assessment Tool for walking and cycling estimate health and economic impacts of increased walking and cycling.

Emergency Preparedness and Response

Table A27. Does your country have plans and procedures for weather and climate-related disaster preparedness, response and recovery? (only 2023).

Region	Yes n (% sub-region)	Under Development n (% sub-region)	No n (% sub-region)	Unknown n (% sub-region)	No response n (% sub-region)
Car (n= 14)	10 (74.4)	1 (7.1)	0 (0)	0 (0)	3 (21.4)
CA (n=9)	6 (66.7)	0 (0)	1 (11.1)	0 (0)	2 (22.2)
SA (n=10)	6 (60)	0 (0)	1 (10)	1(10)	2 (20)

Research and Capacity building

Table A28. Have Ministry of Health staff received any training on the linkages between climate change and health over the past two years? For example, through the PAHO/WHO Virtual Course on Health and Climate Change (only 2023).

Region	Yes<br>n (% sub-region)	No<br>n (% sub-region)	Unknown<br>n (% sub-region)	No response<br>n (% sub-region)
Car (n= 14)	10 (71.4)	0 (0)	1 (7.1))	3 (21.4)
CA (n=9)	4 (44.4)	1 (11.1)	2 (22.2)	2 (22.2)
SA (n=10)	6 (60)	2 (20)	0 (0)	2 (20)

Table A29. If yes, please indicate the category of personnel who have received training on climate change and health. Please select all that apply (only 2023).

Region	Yes<br>n (% sub-region)	No response<br>n (% sub-region)
**Environmental Health Personnel**		
Car (n= 14)	7 (50)	7 (50)
CA (n=9)	4 (44.4)	5 (55.6)
SA (n=10)	6 (60)	4 (40)
**Epidemiology/Surveillance Personnel**		
Car (n= 14)	4 (28.6)	10 (71.4)
CA (n=9)	2 (22.2)	7 (77.8)
SA (n=10)	5 (50)	5 (50)
**Health Emergency Personnel**		
Car (n= 14)	2 (14.3)	12 (85.7)
CA (n=9)	2 (22.2)	7 (77.8)
SA (n=10)	5 (50)	5 (50)
**Health Personnel (e.g., medical doctors, nurses)**		
Car (n= 14)	2 (14.3)	12 (85.7)
CA (n=9)	0 (0)	9 (100)
SA (n=10)	4 (40)	6 (60)
**Planning Personnel**		
Car (n= 14)	2 (14.3)	12 (85.7)
CA (n=9)	0 (0)	9 (100)
SA (n=10)	2 (20)	8 (80)
